# The influence of baseline characteristics on the efficacy of immune checkpoint inhibitors for advanced lung cancer: A systematic review and meta-analysis

**DOI:** 10.3389/fphar.2022.956788

**Published:** 2022-09-09

**Authors:** Qionghua Xiao, Xiaolin Yu, Zhihao Shuai, Ting Yao, Xiaohua Yang, Yanxia Zhang

**Affiliations:** ^1^ Graduate School, Beijing University of Chinese Medicine, Beijing, China; ^2^ The 2nd Department of Pulmonary Disease in Traditional Chinese Medicine (TCM), China-Japan Friendship Hospital, Beijing, China; ^3^ Department of Respiratory, Dongfang Hospital, Beijing University of Chinese Medicine, Beijing, China

**Keywords:** baseline characteristic, immune checkpoint inhibitor, lung cancer, efficacy, meta-analysis

## Abstract

**Purpose:** To investigate the impact of different baseline characteristics on the efficacy of immune checkpoint inhibitors (ICIs) for advanced lung cancer.

**Methods:** In order to identify eligible randomized controlled trials (RCTs), a systematic search was conducted in PubMed, Embase, Cochrane Library, Web of Science, and Scopus databases. The primary outcomes were hazard ratios (HRs) and 95% confidence intervals (CIs) for overall survival (OS). To explore the potential interaction during the administration of ICI, patients were stratified by baseline characteristics.

**Results:** The meta-analysis included 24 RCTs. ① Compared with non-ICI therapy, patients with lung cancer benefitted more from immunotherapy (HR, 0.78; *p* < 0.0001). ② Patients without liver metastases could get more survival benefits than those with liver metastases (HR, 1.20; *p* = 0.0139). Similar outcomes were also observed in the following subgroups: small-cell lung cancer (HR, 1.20; *p* = 0.0433), subsequent line (HR, 1.40; *p* = 0.0147), and ICI monotherapy (HR, 1.40; *p* = 0.0147). ③ Subgroup analysis showed that tumor type affected the efficacy of immunotherapy in patients with brain metastases (HR, 0.72 vs. 1.41; interaction, *p* < 0.01). Among patients with smoking history (HR, 0.87 vs. 1.23; interaction, *p* = 0.05) and brain metastases (HR, 0.69 vs. 1.21; interaction, *p* = 0.05), the type of therapy (i.e., monotherapy or combination therapy) had potential influences on the efficacy of immunotherapy.

**Conclusion:** Some critical baseline characteristics could indicate the efficacy of ICI therapy. Liver metastasis status could predict the efficacy of ICI therapy for lung cancer. Compared with small-cell lung cancer, patients with brain metastases might have durable OS in non-small-cell lung cancer. The smoking history or brain metastasis status of patients could indicate the potential clinical benefits of monotherapy or combination therapy.

## 1 Introduction

Lung cancer is a major threat to people’s health. It is the main cause of cancer death (Siegel et al., 2022). In the past decades, chemotherapy was the main therapy for advanced cancer. However, patients who underwent chemotherapy had a poor prognosis (Sibiya et al., 2019). Although new molecular targeted therapy has improved the treatment of lung cancer, only patients with corresponding genetic mutations can benefit from this therapy (Lee et al., 2018), and drug resistance during the therapeutic process constitutes a pending challenge (Song et al., 2019).

The emergence of immunotherapy improves the treatment mode for lung cancer (Xu et al., 2021). Immune checkpoint inhibitors (ICIs) can block the pathway of cytotoxic T lymphocyte-associated protein-4 (CTLA-4) or programmed death-1 (PD-1). It can enhance T-cell immune responses to prevent the immune escape of tumor cells (Pardoll, 2012). Previous studies have proved a promising survival benefit of immunotherapy for patients with lung cancer (Brahmer et al., 2015; Fehrenbacher et al., 2016; Sugawara et al., 2021). However, only a minority of patients get durable survival from immunotherapy (Hegde and Chen, 2020). Meanwhile, due to the high cost of immunotherapy (Yong et al., 2022) and immune-related adverse reactions (Yu et al., 2021), it is necessary to standardize the application of ICIs. Appropriate baseline characteristics contribute to implementing rational therapeutic strategies. Furthermore, it is conducive to identifying patients suitable for immunotherapy and achieving precise treatment for lung cancer.

In some randomized control trials (RCTs) with prespecified subgroups, the efficacy of immunotherapy varied among patients with different baseline characteristics such as age, gender, and smoking status. For example, in the CheckMate 227 trial (Hellmann et al., 2019), nivolumab combined with ipilimumab significantly improved survival in smoking patients and patients without liver metastases but not in non-smokers and patients with liver metastases. In the CheckMate 057 trial (Borghaei et al., 2015), nivolumab prolonged patients’ overall survival (OS) in second-line therapy and negative epidermal growth factor receptor (EGFR) mutation status. The STIMULI trial (Peters et al., 2022) discovered that no survival benefit of ICIs was observed in male patients, current smoking patients, and 0-point patients in the performance status (PS) of the Eastern Cooperative Oncology Group (ECOG). Therefore, in order to screen the predictors of efficacy of ICIs for lung cancer, it is necessary to conduct pooled analysis of relative RCTs. Recent research (Yan et al., 2020; Wang et al., 2021; Xue et al., 2021) has reported the association between unique baseline characteristics and the efficacy of ICIs for lung cancer. To further investigate the potential influence of baseline characteristics on efficacy, we conducted a comprehensive study in this field.

In this meta-analysis, the efficacy of immunotherapy and non-ICI therapy in different baseline characteristics was systematically assessed. Meanwhile, we also calculated the interaction of baseline characteristics that might influence the efficacy. Moreover, we summarized the immune-related adverse events (irAEs) of immunotherapy for lung cancer and illustrated them with a table in the Results section. This study involved a total of 11 systems and contained the incidence and common disease severity. Then, we expounded the importance of standardized and precise medication of ICIs.

## 2 Materials and methods

### 2.1 Protocol and registration

This review followed the Preferred Reporting Items for Systematic Reviews and Meta-Analysis (PRISMA) guidelines (Moher et al., 2009) (Supplementary Table S1). The research protocol has been submitted to the PROSPERO platform (https://www.crd.york.ac.uk/prospero/) (Registration No.: CRD42022326099).

### 2.2 Search strategy

PubMed, Embase, Cochrane Library, Web of Science, and Scopus databases were searched for phase 2 or 3 RCTs up to 10 February 2022. Conference proceedings of medical societies, such as the European Society of Medical Oncology and the American Society of Clinical Oncology, were also reviewed. Two authors (Xiao and Yu) independently completed the retrieval of the literature. The search terms used are as follows: “Nivolumab,” “Pembrolizumab,” “Cemiplimab,” “Atezolizumab,” “Durvalumab,” “Avelumab,” “Ipilimumab,” “Tremelimumab,” “Programmed Cell Death 1 Receptor (PD-1),” “Programmed Cell Death 1 Ligand 1 Protein (PD-L1),” “Cytotoxic T-Lymphocyte Associated Antigen 4 (CTLA-4),” “immune checkpoint inhibitor,” “Lung Cancer,” and “randomized controlled trial.” The details of the search strategy are provided in Supplementary Table S2.

### 2.3 Inclusion and exclusion criteria

The studies were included in this meta-analysis when they met the following inclusion criteria: 1) Population: patients diagnosed as lung cancer. 2) Intervention: ICI monotherapy or combined with other therapies (i.e., another immunotherapy, chemotherapy, target therapy, or radiotherapy). 3) Comparison: chemotherapy or placebo. 4) Outcome: hazard ratios (HRs) and 95% confidence intervals (CIs) for OS. 5) Study design: phase 2 or 3 RCTs.

The exclusion criteria of this study were as follows: 1) observational studies, 2) single-arm trials, 3) studies with duplicated data in the same population, 4) conference articles for which the full text is not available, and 5) articles in a language other than English.

### 2.4 Data extraction and quality assessment

Data extraction was carried out by two reviewers (Shuai and Yao) according to the predetermined list of information. All of the disagreements were resolved through consultation with all investigators. The basic information of the study was extracted, including first author, publication year, trial name, types of lung cancer, study phase, line of therapy, PD-L1 expression, therapeutic drug, number of patients, overall HRs, and HRs for each baseline characteristic.

The risk of bias was evaluated according to seven aspects (Higgins et al., 2011): random sequence generation (selection bias), allocation concealment (selection bias), blinding of participants and personnel (performance bias), blinding of outcome assessment (detection bias), incomplete outcome data (attrition bias), selective reporting (reporting bias), and other bias.

### 2.5 Statistical analysis

The clinical heterogeneity of the included studies was assessed by the characteristics of populations, interventions, control group, outcomes, and study design. All statistical analyses were conducted by the “meta” package in R version 4.1.3 (Balduzzi et al., 2019). The HRs and 95% CIs for OS in patients stratified by each baseline characteristic were collected from original studies. We used the standard Cochran’s Q test and I^2^ statistics to evaluate the heterogeneity of included studies (Higgins et al., 2003). I^2^ > 40% or *p* < 0.1 represented significant heterogeneity, and a random-effects model was chosen. Otherwise, a fixed-effects model was used (Sethi et al., 2019). In order to assess the difference in immunotherapy efficacy among patients stratified by baseline characteristics, we used the following methods: first, the inverse variance method was applied to calculate pooled HRs for OS in patients. Then, we calculated interaction HRs for baseline characteristics (Altman and Bland, 2003) and integrated these data. Subgroup analysis was also performed in this review. The subgroups include types of lung cancer, lines of treatment, and types of therapy. The publication bias was evaluated using the funnel plot, Egger’s test, and Begg’s test.

## 3 Results

### 3.1 Study selection

**FIGURE 1:**
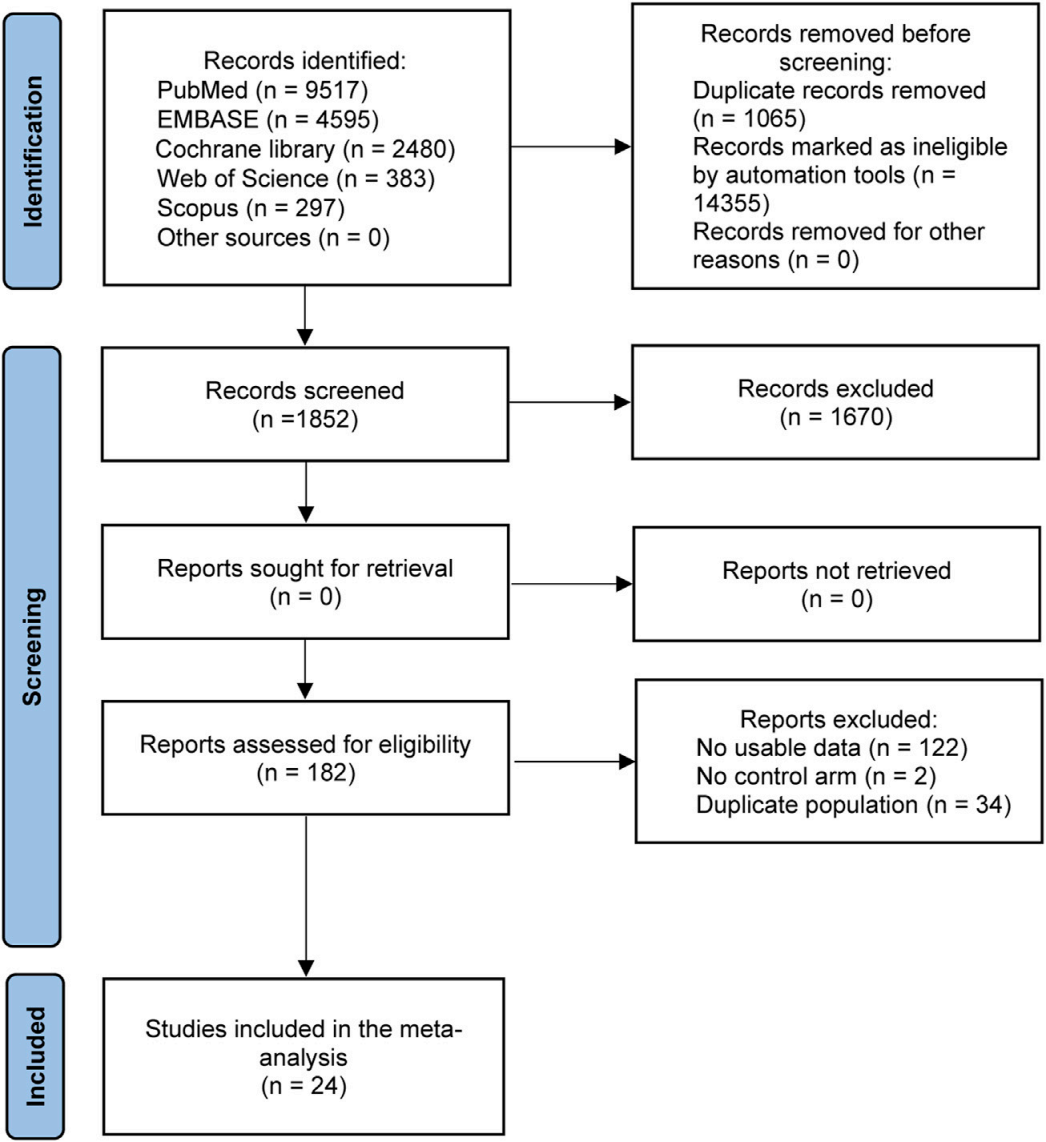
Flow chart diagram of study selection.

The flow chart of literature selection is shown in Figure 1. In total, 17,272 literature works were identified through searching PubMed, Embase, Cochrane library, Web of Science, and Scopus databases and conference proceedings up to 10 February 2022. After removing duplicate studies, filtering titles and abstracts, and reviewing the full texts, 24 studies with 15,628 patients were ultimately included in this meta-analysis (Borghaei et al., 2015; Reck et al., 2016; Carbone et al., 2017; Govindan et al., 2017; Rittmeyer et al., 2017; Paz-Ares et al., 2018; Hellmann et al., 2019; Reck et al., 2019; West et al., 2019; Rudin et al., 2020; Goldman et al., 2021; Herbst et al., 2021; Liu et al., 2021; Nishio et al., 2021; Owonikoko et al., 2021; Park et al., 2021; Reck et al., 2021; Rodriguez-Abreu et al., 2021; Sezer et al., 2021; Socinski et al., 2021; Spigel et al., 2021; Wu et al., 2021; Peters et al., 2022; Spigel et al., 2022).

### 3.2 Study characteristics

The study characteristics of 24 RCTs are summarized in Table 1. All eligible studies were published between 2015 and 2022. Among the 24 studies, a total of 17 studies researched on non-small-cell lung cancer (NSCLC) and seven studies researched on small-cell lung cancer (SCLC). In all the included trials, there were 16 trials that focused on first-line therapy of patients with lung cancer and 8 trials focused on subsequent-line therapy. All of the included studies were comparisons between ICI-based therapy and non-ICI therapy. A total of 12 trials used ICI monotherapy, and nine trials used ICI combined with chemotherapy, and only three trials used ICI combined with ICI. Overall HRs were collected from each study.

**TABLE 1:** Characteristics of included studies.

								Patients (No.)		
Author (year)	Study name	NCT number	Tumor type	Phase	Line	PD-L1 level	Treatment	Int	Con	Overall HR (95%CI)
Paz-Ares et al. (2018)	KEYNOTE-407	NCT02775435	NSCLC	3	1	Any level	Pembrolizumab	278	281	0.64 (0.49, 0.85)
Borghaei et al. (2015)	CheckMate 057	NCT01673867	NSCLC	3	>1	Any level	Nivolumab	292	290	0.75 (0.62, 0.91)
Sezer et al. (2021)	EMPOWER-Lung 1	NCT03088540	NSCLC	3	1	≥50%	Cemiplimab	283	280	0.57 (0.42, 0.77)
Carbone et al. (2017)	CheckMate 026	NCT02041533	NSCLC	3	1	≥5%	Nivolumab	271	270	1.08 (0.87, 1.34)
Reck et al. (2019)	KEYNOTE-024	NCT02142738	NSCLC	3	1	≥50%	Pembrolizumab	154	151	0.63 (0.47, 0.86)
Wu et al. (2021)	KEYNOTE-042 China study	NCT02220894	NSCLC	3	1	Any level	Pembrolizumab	128	134	0.67 (0.50, 0.89)
Park et al. (2021)	JAVELIN Lung 200	NCT02395172	NSCLC	3	>1	NR	Avelumab	264	265	0.87 (0.72, 1.06)
Spigel et al. (2022)	PACIFIC	NCT02125461	NSCLC	3	>1	Any level	Durvalumab	476	237	0.72 (0.59, 0.87)
Rittmeyer et al. (2017)	OAK study	NCT02008227	NSCLC	3	>1	Any level	Atezolizumab	613	612	0.73 (0.62, 0.87)
Herbst et al. (2021)	KEYNOTE-010	NCT01905657	NSCLC	3	>1	Any level	Pembrolizumab	690	343	0.70 (0.61, 0.80)
Govindan et al. (2017)	NR	NCT01285609	NSCLC	3	1	NR	Ipilimumab+ chemotherapy	479	477	0.91 (0.77, 1.07)
West et al. (2019)	IMpower130	NCT02367781	NSCLC	3	1	Any level	Atezolizumab+ chemotherapy	451	228	0.79 (0.64, 0.98)
Nishio et al. (2021)	IMpower132	NCT02657434	NSCLC	3	1	Any level	Atezolizumab+ chemotherapy	292	286	0.86 (0.71, 1.06)
Rodriguez-Abreu et al. (2021)	KEYNOTE-189	NCT02578680	NSCLC	3	1	Any level	Pembrolizumab+ chemotherapy	410	206	0.56 (0.46, 0.69)
Socinski et al. (2021)	IMpower150	NCT02366143	NSCLC	3	1	Any level	Atezolizumab+ chemotherapy	400	400	0.80 (0.67, 0.95)
Hellmann et al. (2019)	CheckMate 227	NCT02477826	NSCLC	3	1	Any level	Nivolumab+ ipilimumab	583	583	0.79 (0.65, 0.96)
Reck et al. (2021)	CheckMate 9LA	NCT03215706	NSCLC	3	1	Any level	Nivolumab+ ipilimumab+ chemotherapy	361	358	0.73 (0.61, 0.87)
Spigel et al. (2021)	CheckMate 331	NCT02481830	SCLC	3	>1	NR	Nivolumab	284	285	0.87 (0.73, 1.05)
Owonikoko et al. (2021)	CheckMate 451	NCT02538666	SCLC	3	>1	any level	Nivolumab	280	275	0.83 (0.68, 1.01)
Goldman et al. (2021)	CASPIAN	NCT03043872	SCLC	3	1	NR	Durvalumab+ chemotherapy	268	269	0.75 (0.62, 0.91)
Liu et al. (2021)	IMpower133	NCT02763579	SCLC	3	1	any level	Atezolizumab+ chemotherapy	201	202	0.76 (0.60, 0.95)
Rudin et al. (2020)	KEYNOTE-604	NCT03066778	SCLC	3	1	NR	Pembrolizumab+ chemotherapy	228	225	0.80 (0.64, 0.98)
Reck et al. (2016)	NR	NCT01450761	SCLC	3	1	NR	Ipilimumab+ chemotherapy	566	566	0.94 (0.81, 1.09)
Peters et al. (2022)	STIMULI trial	NCT02046733	SCLC	2	>1	NR	Nivolumab+ ipilimumab	78	75	0.94 (0.59, 1.50)

Abbreviations: NCT, national clinical trial; PD-L1, programmed cell death 1 ligand 1; Int, intervention group; Con, control group; HR, hazard ratio; CI, confidence interval; NR, not reported; NSCLC, non-small-cell lung cancer; SCLC, small-cell lung cancer.

### 3.3 Risk of bias assessment

The risk of bias assessment is provided in Supplementary Table S3. In all trials, the risk of bias was low in terms of random sequence generation, completeness of outcome data, selective reporting, and other biases. Because of the open-label design, some trials may increase the risk of bias. In general, the quality of these trials was satisfactory.

### 3.4 Pooled hazard ratios of patients with lung cancer

**FIGURE 2:**
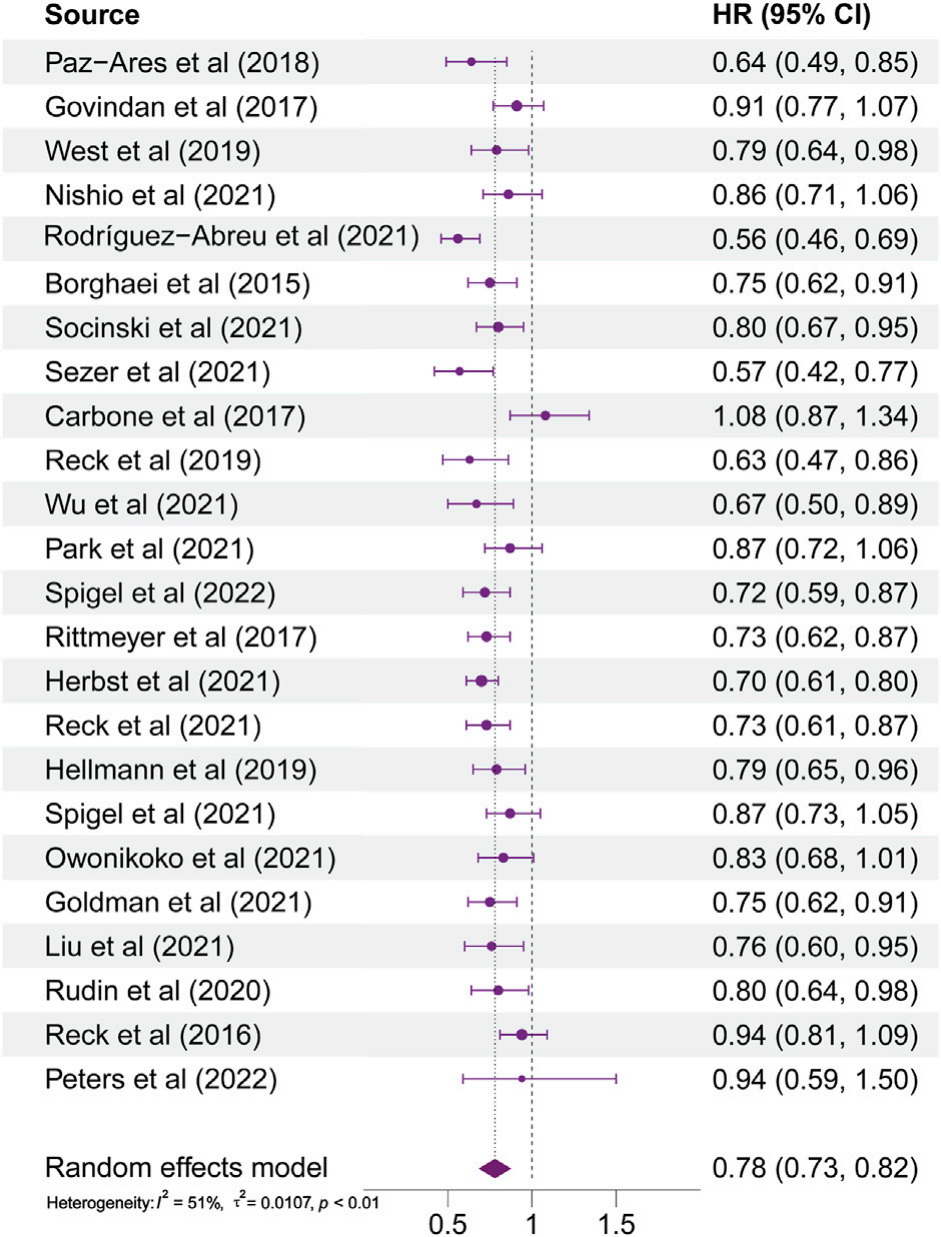
Forest plot of all patients’ hazard ratios for overall survival.

As illustrated in Figure 2, HRs of almost all studies were on the left side of the axis in the forest plots. It indicated that ICI therapy had more benefits than non-ICI therapy. However, the HR reported by Carbone et al. (2017) trended to the right side of the axis. It represented that patients administered with ICI therapy did not gain a more lasting survival than those in the chemotherapy group. The range of 95% CI reported by Peters et al. (2022) illustrated that the efficacy of ICI therapy for different patients varies greatly in this study. In general, patients with lung cancer using ICI therapy received more significant benefits than those using non-ICI therapy (HR, 0.78; 95% CI, 0.73–0.82; *p* < 0.0001). Subgroup analyses were also conducted according to patients’ different baseline characteristics. The results showed that the source of heterogeneity was liver metastases status and PD-L1 expression level.

### 3.5 Pooled hazard ratios and interaction hazard ratios of patients stratified by baseline characteristics

**FIGURE 3:**
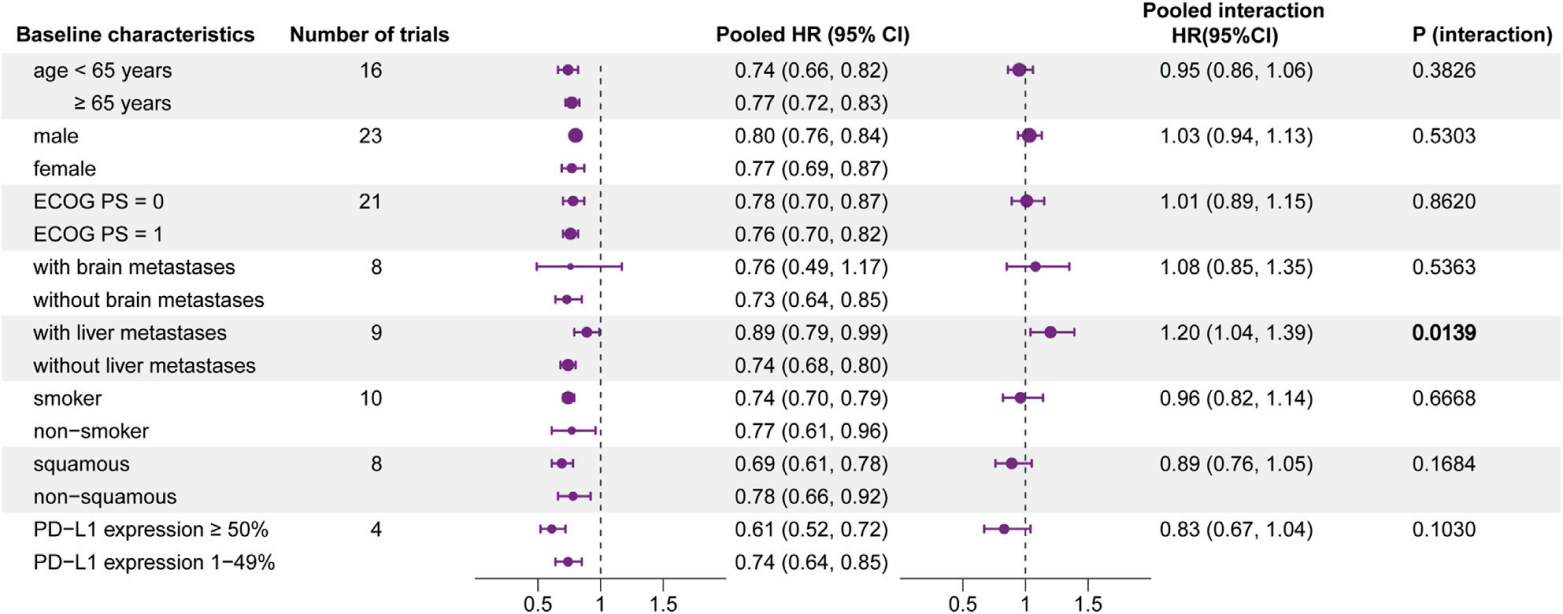
Forest plot of hazard ratios for overall survival according to baseline characteristics. Left forest plot: hazard ratios of overall survival for patients assigned to the intervention group, compared with those assigned to the control group, stratified by each baseline characteristic. Right forest plot: interaction between immunotherapy efficacy and each baseline characteristic.

The pooled HRs and potential interactions are shown in Figure 3. The left forest plot represented pooled HRs (ICIs vs. non-ICIs) for each baseline characteristic. In the group of patients with brain metastases, there was no significant difference between ICI therapy and non-ICI therapy (HR, 0.76; 95% CI, 0.49–1.17; *p* = 0.2142). It indicated that patients with brain metastases may receive relatively rare clinical benefits from ICI-based therapy.

The interaction is shown in the right part of Figure 3. It represented the interaction between immunotherapy and each baseline characteristic. The pooled interaction HR between patients with liver metastases and without liver metastases was 1.20 (95% CI, 1.04–1.39; *p* = 0.0139). This illustrated that patients without liver metastases may gain more benefits from ICI therapy. Patients stratified by other baseline characteristics did not show a significant difference in efficacy.

### 3.6 Subgroup analysis

#### 3.6.1 Type of lung cancer

**FIGURE 4:**
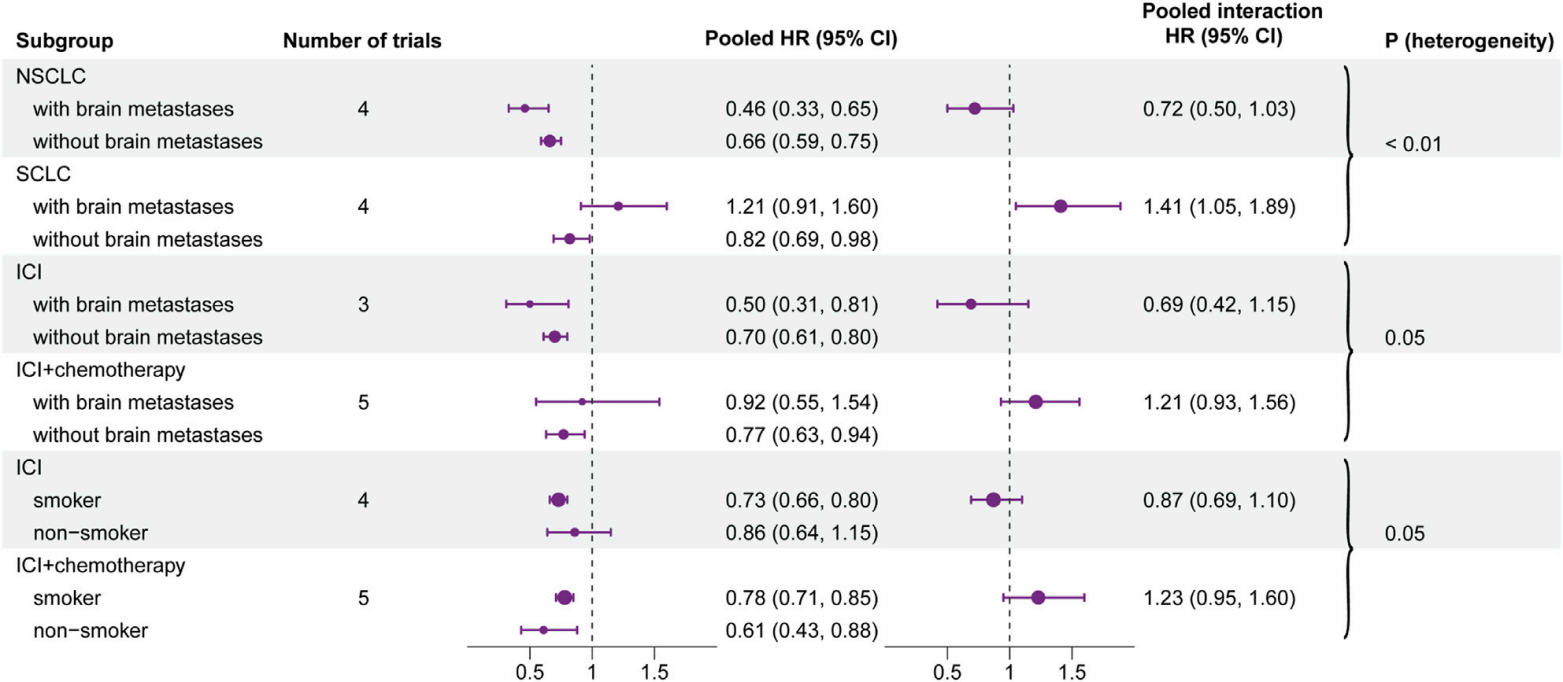
Forest plot of hazard ratios for overall survival according to the positive results of each subgroup. Left forest plot: hazard ratios of overall survival for subgroups and baseline characteristics. Right forest plot: interaction between immunotherapy efficacy and baseline characteristics in different subgroups (i.e., type of therapy and type of cancer).

The pooled interaction in patients with brain metastases or without brain metastases was 0.72 (95% CI, 0.50–1.03) in NSCLC and 1.41 (95% CI, 1.05–1.89) in SCLC. By conducting a heterogeneity test of interaction between subgroups, the results showed that there was significant heterogeneity between NSCLC and SCLC (HR, 0.72 vs. 1.41; interaction, *p* < 0.01) (Figure 4). In other baseline characteristics, no significant difference was observed (Supplementary Figure S1).

#### 3.6.2 Line of treatment

Details on the subgroup of line of treatment are shown in Supplementary Figure S2. In the following baseline characteristics, no difference in interaction in the lines of therapy was observed: age, gender, ECOG PS, liver metastasis status, smoking status, and histological type.

#### 3.6.3 Type of therapy

The pooled interaction in patients with brain metastases or without brain metastases was 0.69 (95% CI, 0.42–1.15) in the group of ICI monotherapy and 1.21 (95% CI, 0.93–1.56) in the group of ICI combined with chemotherapy. Further analyses illustrated that potential heterogeneity existed between ICI monotherapy and combination therapy (HR, 0.69 vs. 1.21; interaction, *p* = 0.05). Similar results were also observed in the smoking status. The pooled interaction between smoking patients and non-smokers was 0.87 (95% CI, 0.69–1.10) in ICI monotherapy and 1.23 (95% CI, 0.95–1.60) in combination therapy. Also, the heterogeneity test demonstrated that there was a significant difference between the two groups (HR, 0.87 vs. 1.23; interaction, *p* = 0.05) (Figure 4). In other baseline characteristics, no obvious heterogeneity was observed (Supplementary Figure S3).

### 3.7 Immune-related adverse events

Common irAEs of immunotherapy for lung cancer are shown in Table 2. A total of 11 systems were involved in this study, and it contained the incidence and common disease severity of irAEs. The prevalent severity of most adverse events was graded 1 and 2, while encephalitis, meningitis, multiple sclerosis, and myelitis were graded 3 and 4. Due to the rare incidence of some irAEs, there is currently no report on their disease severity.

**TABLE 2:** Incidence and disease severity of common irAEs.

System	Common irAE	Incidence	Common disease severity (grade)
Dermatology	Alopecia	28.57%	1/2
	Rash	10%	1/2
	Pruritus	9.46%	1/2
Endocrine	Hypothyroidism	6.54%	1/2
	Hyperthyroidism	2.89%	1/2
	Primary adrenal insufficiency	0.50%	1/2
	Type 1 diabetes	0.15%	1/2
	Hypophysitis	0.10%	1/2
Gastrointestinal	Nausea	32.43%	1/2
	Colitis	20%	1/2
	Diarrhea	8%	1/2
Hepatic	Hepatitis	7.60%	1/2
Pulmonary	Pneumonitis	12.20%	1/2
	Sarcoidosis	7%	1/2
	Interstitial lung disease	5%	1/2
Renal	Acute kidney injury	2%	1/2
Musculoskeletal	Inflammatory arthritis	12%	1/2
	Myositis	12%	1/2
Cardiac and vascular	Myocarditis	2.40%	NR
	Pericarditis/pericardial effusion	1.90%	NR
	Vasculitis	0.63%	NR
Nervous	Myositis	3%	1/2
	Peripheral neuropathy	1.20%	1/2
	Guillain–Barre syndrome	0.30%	1/2
	Encephalitis	0.16%	3/4
	Meningitis	0.13%	3/4
	Multiple sclerosis	0.03%	3/4
	Myelitis	<0.01%	3/4
Ocular	Uveitis	0.50%	1/2
Hematology	Neutropenia	0.94%	NR
	Hemolytic anemia	0.60%	NR
	Thrombocytopenia	<0.01%	NR

Abbreviations: irAEs, immune-related adverse events; NR, not reported.

### 3.8 Sensitivity analysis and publication bias

In order to evaluate the influence of each study on overall outcomes, we conducted the sensitivity analysis with both the fixed effects model and random effects model. Similar results were obtained from two models. The absence of each study cannot significantly change the overall values, and it verified the stability of the results in our meta-analysis (Supplementary Figure S4).

We used two methods to assess the publication bias. The *p*-value of Egger’s test was 0.3003 and that of Begg’s test was 0.2145. Evidence of publication bias was not detected by the funnel plot (Supplementary Figure S5).

## 4 Discussion

Despite considerable breakthroughs in immunotherapy in the treatment of lung cancer, only few patients benefit from ICIs. Therefore, it makes sense to explore appropriate predictors for suitable patients. Previous studies (Yan et al., 2020; Wang et al., 2021; Xue et al., 2021) found that baseline characteristics (i.e., age, gender, and brain metastases, etc.) could influence the efficacy of ICIs on lung cancer. In practical clinical applications, the interaction among these single characteristics might influence the final responses of the immunotherapy. From a whole perspective, the interaction should be given more importance. Therefore, in order to provide references for clinical decisions, this review analyzed the impact of common baseline characteristics on efficacy. As far as we know, this is the first meta-analysis to investigate the effect of comprehensive baseline characteristics on the efficacy of ICIs for lung cancer.

In general, patients who received ICI-based therapy acquired better survival benefits than those who received non-ICI therapy. However, the HR reported by Carbone et al. (2017). trended to the right side of the axis. The reason was unbalanced baseline characteristics between the two groups. Disease characteristics associated with better prognosis are favored by the chemotherapy group. Because of the discontinuation of some patients, the range of 95% CI reported by Peters et al. (2022) illustrated that the efficacy of ICI therapy for different patients varies greatly in this study.

The statistical heterogeneity of all patients’ HRs for OS among included studies was detected (I^2^ = 51%; *p* < 0.01), and we performed this meta-analysis via the random effects model. Statistical heterogeneity was the result of the synergy of clinical and methodological diversity among studies. Due to differences in terms of the populations, interventions, control group, outcome indicators, and study design, there may be clinical heterogeneity among studies. We carefully assessed the differences in these aspects and conducted subgroup analyses to explore the source of heterogeneity. The results showed that the source of heterogeneity was liver metastasis status and PD-L1 expression level. Differences in the study design may lead to methodological heterogeneity, which included, randomization, application of blinding, allocation concealment, completeness of outcome reporting, and rigorousness of statistical analysis. In this meta-analysis, the study design and research quality of the included trials did not show obvious differences.

Although current genetic tests and immunohistochemical tests can direct the application of ICIs in most cases, some baseline characteristics may have potentially directive functions in the choice of ICIs. Liver metastases were potentially related to the poor prognosis of immunotherapy on lung cancer according to the results of our meta-analysis. Immunotherapy in patients without liver metastases might result in a more lasting OS than those with liver metastases. Similar results were also observed in other subgroups (i.e., pathological type, subsequent line, and ICI monotherapy). It was consistent with other reports (Tumeh et al., 2017; Tournoy et al., 2018). The incidence of liver metastases of lung cancer is rare in clinical real-world settings. In the included studies of the liver metastases group, the sample size of patients with or without liver metastases was 1,346 and 3,807, respectively. The incidence of liver metastases in patients with lung cancer was 26.12%. Since we included patients with advanced lung cancer, the sample size of patients with liver metastases would be relatively large. This value did not represent the incidence in the general population. Due to the limitation of sample size, we tentatively drew this conclusion. It is necessary to increase the sample size to further explore the influence of liver metastasis status on immunotherapy for lung cancer.

The liver induces immunotolerance by multiple mechanisms, including poor activation of CD4^+^ T cells (Wang and Livingstone, 2003), incomplete activation of CD8^+^ T cells (Limmer et al., 2000), and the apoptosis of activated CD8^+^ T cells (Crispe, 2003). The tolerable hepatic microenvironment may interfere with the reaction of ICIs in patients with liver metastasis. Meanwhile, CD8^+^ T cells are associated with the reaction of PD-1 inhibitors. CD8^+^ T cells are depleted in patients with liver metastases. Therefore, liver metastasis status has an impact on the response of ICIs (Tumeh et al., 2017). Recently, Wu et al. (2022) found that immunosuppressive cells, such as MRC1 CCL18 M2-like macrophages, showed an upward trend in the liver metastasis sites. MRC1 CCL18 M2-like macrophages displayed enhanced metabolic activity in all myeloid cells, resulting in a suppressed immune state and reduced efficacy of immunotherapy. The neoadjuvant chemotherapy mentioned in this study may offer new treatment ideas for patients with liver metastases. The study by Liu et al. (2022) bridged immune phenotypes of primary and metastatic tumors. They found that for myeloid cells, M-type SPP1 macrophages were predominant in liver metastasis, leading to the immunosuppressive state.

In addition, we conducted heterogeneous tests for the interaction of each baseline characteristic in every subgroup. The result showed that the efficacy of patients with brain metastases exhibited significant heterogeneity between NSCLC and SCLC (*p* < 0.01). It illustrated that tumor types may influence the efficacy of immunotherapy on patients with brain metastases. This phenomenon may be related to the PD-L1 expression level. A higher PD-L1 expression level has been proved to be associated with enhanced efficacy of immunotherapy in NSCLC (Mansour et al., 2022). In our review, patients with brain metastases who received ICI therapy got less benefits than patients who received non-ICI therapy in SCLC. For asymptomatic patients with brain metastases in extensive-stage SCLC, National Comprehensive Cancer Network (NCCN) guidelines (Ganti et al., 2021) recommended the systemic therapy of atezolizumab or durvalumab combined with chemotherapy. The ICI drugs used in two of four included studies were beyond the recommendation of the NCCN guidelines, and corresponding research was still under investigation. The efficacy of ICIs for SCLC still needs further exploration.

In the subgroup of type of treatment, the efficacy of patients with brain metastases between ICI monotherapy and combination therapy had potential heterogeneity. The same research results were also observed in smoking status. It indicated that the type of treatment may affect the efficacy of immunotherapy for patients with brain metastases and smoking status. In our meta-analysis, compared with ICI monotherapy, patients with brain metastases who received combination therapy showed less clinical benefits. In our included articles, three of four studies in NSCLC applied ICI monotherapy, and all four studies in SCLC applied ICI combined with chemotherapy. In the aforementioned discussion, we discussed the reason for the poor efficacy of ICIs for SCLC in this review. So patients with brain metastases who received combination therapy gained less benefits from ICI therapy.

Moreover, our meta-analysis discovered that patients with smoking history benefitted more from ICI monotherapy, while non-smokers obtained more benefits from combination therapy. On the one hand, the study by Yang et al. (2021) pointed out that smoking may increase tumor mutation burden (TMB) and microsatellite instability (MSI). This mechanism may improve the reaction of immunotherapy. On the other hand, from the point of pathological type, patients with smoking history took higher risks on suffering from squamous cell carcinoma (Park et al., 2010), while non-smokers may have higher genetic susceptibility to adenocarcinoma (Fu et al., 2022). The research by Tian et al. (2021) indicated that patients with squamous cell carcinoma may obtain more lasting OS from ICIs than those with adenocarcinoma. More functional tumor-infiltrating lymphocytes and chemokines in the tumor microenvironment are present in squamous NSCLC.

Our study found that gender is not associated with the efficacy of immunotherapy for lung cancer. This is different from the results of previous studies (Jang et al., 2021; Wu et al., 2018b). We found that the reason for the different results among the studies may be due to different patient populations. The study by Jang et al. (2021) included patients with melanoma; Wu et al. (2018b) included patients with various tumor types, while our study just included patients with lung cancer. The efficacy of immunotherapy varies by tumor types.

In terms of the comparison between PD-1 and PD-L1 agents, no statistically significant difference was found in the efficacy between PD-1 and PD-L1 agents for patients with lung cancer (HR, 0.73 vs. 0.78; *p* = 0.34). This result is different from the previous literature (Duan et al., 2020; Wu et al., 2018a). The possible reason may be due to the different patient populations. The study by Duan et al. (2020) included patients with solid tumors; Wu et al. (2018a) included patients with NSCLC, while our study included patients with lung cancer. Immunotherapy responds differently in different tumor types.

A previous study indicated that about 40% of patients using immunotherapy may experience irAEs (D'Souza et al., 2021). The study by Garrett et al. (2020) also reported the incidence and disease severity of dermatological toxicities and provided a rational strategy for the management of irAEs. Our study summarized the frequent irAEs of immunotherapy for lung cancer. We found that irAEs are difficult to avoid in the process of immunotherapy. Individualized precision medicine may have the potential effects on balancing the efficacy of immunotherapy and the occurrence of irAEs.

Empirical targeted therapy is less dependent on emerging technologies. However, only a minority of patients with cancer could get durable survival from it. It is necessary to determine the most suitable treatment plan for different patients. Precision medicine fully considers the individual heterogeneity of patients. In this way, the treatment plan will take into account the best treatment effect and the best medical cost-effectiveness ratio. Therefore, precision medicine is becoming the trend in cancer treatment.

However, there are several potential limitations to this study. First, HRs for OS in patients with various baseline characteristics were provided by different clinical trials and various institutions. It may lead to inaccurate data collection. Second, owing to the fact that we just focussed on RCTs, the data of patients who did not meet the inclusion criteria were missing. Third, although our review covered the effect of common baseline characteristics on the efficacy of ICIs for lung cancer, due to insufficient data on other baseline characteristics, there may exist other confounding factors which might affect the efficacy, such as region and disease severity.

## 5 Conclusion

Current data indicated that ICI-based therapy improved the OS of most patients stratified by different baseline characteristics compared with non-ICI therapy. Liver metastasis status would influence the final efficacy of ICIs, and other baseline characteristics might be independent of it. Through subgroup analysis, compared with small-cell lung cancer, patients with brain metastases might have a more durable OS in non-small-cell lung cancer. The smoking history or brain metastasis status of patients could indicate the potential clinical benefits of monotherapy or combination therapy.

## Data Availability

The original contributions presented in the study are included in the article/Supplementary Material; further inquiries can be directed to the corresponding author.

## References

[B1] Altman D. G. Bland J. M. (2003). Interaction revisited: the difference between two estimates. BMJ 326 (7382), 219. 10.1136/bmj.326.7382.219 12543843 PMC1125071

[B2] Balduzzi S. Rucker G. Schwarzer G. (2019). How to perform a meta-analysis with R: a practical tutorial. Evid. Based. Ment. Health 22 (4), 153–160. 10.1136/ebmental-2019-300117 31563865 PMC10231495

[B3] Borghaei H. Paz-Ares L. Horn L. Spigel D. R. Steins M. Ready N. E. (2015). Nivolumab versus docetaxel in advanced nonsquamous non-small-cell lung cancer. N. Engl. J. Med. 373 (17), 1627–1639. 10.1056/NEJMoa1507643 26412456 PMC5705936

[B4] Brahmer J. Reckamp K. L. Baas P. Crino L. Eberhardt W. E. Poddubskaya E. (2015). Nivolumab versus docetaxel in advanced squamous-cell non-small-cell lung cancer. N. Engl. J. Med. 373 (2), 123–135. 10.1056/NEJMoa1504627 26028407 PMC4681400

[B5] Carbone D. P. Reck M. Paz-Ares L. Creelan B. Horn L. Steins M. (2017). First-line nivolumab in stage IV or recurrent non-small-cell lung cancer. N. Engl. J. Med. 376 (25), 2415–2426. 10.1056/NEJMoa1613493 28636851 PMC6487310

[B6] Crispe I. N. (2003). Hepatic T cells and liver tolerance. Nat. Rev. Immunol. 3 (1), 51–62. 10.1038/nri981 12511875

[B7] D'Souza M. Nielsen D. Svane I. M. Iversen K. Rasmussen P. V. Madelaire C. (2021). The risk of cardiac events in patients receiving immune checkpoint inhibitors: a nationwide Danish study. Eur. Heart J. 42 (16), 1621–1631. 10.1093/eurheartj/ehaa884 33291147

[B8] Duan J. Cui L. Zhao X. Bai H. Cai S. Wang G. (2020). Use of immunotherapy with programmed cell death 1 vs programmed cell death ligand 1 inhibitors in patients with cancer: A systematic review and meta-analysis. JAMA Oncol. 6 (3), 375–384. 10.1001/jamaoncol.2019.5367 31876895 PMC6990765

[B9] Fehrenbacher L. Spira A. Ballinger M. Kowanetz M. Vansteenkiste J. Mazieres J. (2016). Atezolizumab versus docetaxel for patients with previously treated non-small-cell lung cancer (POPLAR): a multicentre, open-label, phase 2 randomised controlled trial. Lancet 387 (10030), 1837–1846. 10.1016/s0140-6736(16)00587-0 26970723

[B10] Fu R. Zhang J. T. Chen R. R. Li H. Tai Z. X. Lin H. X. (2022). Identification of heritable rare variants associated with early-stage lung adenocarcinoma risk. Transl. Lung Cancer Res. 11 (4), 509–522. 10.21037/tlcr-21-789 35529798 PMC9073742

[B11] Ganti A. K. P. Loo B. W. Bassetti M. Blakely C. Chiang A. D'Amico T. A. (2021). Small cell lung cancer, version 2.2022, NCCN clinical practice guidelines in Oncology. J. Natl. Compr. Canc. Netw. 19 (12), 1441–1464. 10.6004/jnccn.2021.0058 34902832 PMC10203822

[B12] Garrett N. da Costa A. C. C. Damiani G. Vasques C. I. (2020). Patients with lung cancer undergoing immune checkpoint inhibitors: A meta-analysis of dermatological toxicities. Crit. Rev. Oncol. Hematol. 152, 102983. 10.1016/j.critrevonc.2020.102983 32570149

[B13] Goldman J. W. Dvorkin M. Chen Y. Reinmuth N. Hotta K. Trukhin D. (2021). Durvalumab, with or without tremelimumab, plus platinum–etoposide versus platinum–etoposide alone in first-line treatment of extensive-stage small-cell lung cancer (CASPIAN): updated results from a randomised, controlled, open-label, phase 3 trial. Lancet. Oncol. 22 (1), 51–65. 10.1016/s1470-2045(20)30539-8 33285097

[B14] Govindan R. Szczesna A. Ahn M. J. Schneider C. P. Gonzalez Mella P. F. Barlesi F. (2017). Phase III trial of ipilimumab combined with paclitaxel and carboplatin in advanced squamous non-small-cell lung cancer. J. Clin. Oncol. 35 (30), 3449–3457. 10.1200/JCO.2016.71.7629 28854067

[B15] Hegde P. S. Chen D. S. (2020). Top 10 challenges in cancer immunotherapy. Immunity 52 (1), 17–35. 10.1016/j.immuni.2019.12.011 31940268

[B16] Hellmann M. D. Paz-Ares L. Bernabe Caro R. Zurawski B. Kim S. W. Carcereny Costa E. (2019). Nivolumab plus ipilimumab in advanced non-small-cell lung cancer. N. Engl. J. Med. 381 (21), 2020–2031. 10.1056/NEJMoa1910231 31562796

[B17] Herbst R. S. Garon E. B. Kim D. W. Cho B. C. Gervais R. Perez-Gracia J. L. (2021). Five year survival update from KEYNOTE-010: Pembrolizumab versus docetaxel for previously treated, programmed death-ligand 1-positive advanced NSCLC. J. Thorac. Oncol. 16 (10), 1718–1732. 10.1016/j.jtho.2021.05.001 34048946

[B18] Higgins J. P. Thompson S. G. Deeks J. J. Altman D. G. (2003). Measuring inconsistency in meta-analyses. BMJ 327 (7414), 557–560. 10.1136/bmj.327.7414.557 12958120 PMC192859

[B19] Higgins J. P. Altman D. G. Gotzsche P. C. Juni P. Moher D. Oxman A. D. (2011). The Cochrane Collaboration's tool for assessing risk of bias in randomised trials. BMJ 343, d5928. 10.1136/bmj.d5928 22008217 PMC3196245

[B20] Jang S. R. Nikita N. Banks J. Keith S. W. Johnson J. M. Wilson M. (2021). Association between sex and immune checkpoint inhibitor outcomes for patients with melanoma. JAMA Netw. Open 4 (12), e2136823. 10.1001/jamanetworkopen.2021.36823 34854905 PMC8640892

[B21] Lee Y. T. Tan Y. J. Oon C. E. (2018). Molecular targeted therapy: treating cancer with specificity. Eur. J. Pharmacol. 834, 188–196. 10.1016/j.ejphar.2018.07.034 30031797

[B22] Limmer A. Ohl J. Kurts C. Ljunggren H. G. Reiss Y. Groettrup M. (2000). Efficient presentation of exogenous antigen by liver endothelial cells to CD8+ T cells results in antigen-specific T-cell tolerance. Nat. Med. 6 (12), 1348–1354. 10.1038/82161 11100119

[B23] Liu S. V. Reck M. Mansfield A. S. Mok T. Scherpereel A. Reinmuth N. (2021). Updated overall survival and PD-L1 subgroup Analysis of patients with extensive-stage small-cell lung cancer treated with atezolizumab, carboplatin, and etoposide (IMpower133). J. Clin. Oncol. 39 (6), 619–630. 10.1200/JCO.20.01055 33439693 PMC8078320

[B24] Liu Y. Zhang Q. Xing B. Luo N. Gao R. Yu K. (2022). Immune phenotypic linkage between colorectal cancer and liver metastasis. Cancer Cell 40 (4), 424–437.e5. 10.1016/j.ccell.2022.02.013 35303421

[B25] Mansour M. S. I. Malmros K. Mager U. Ericson Lindquist K. Hejny K. Holmgren B. (2022). PD-L1 expression in non-small cell lung cancer specimens: Association with clinicopathological factors and molecular alterations. Int. J. Mol. Sci. 23 (9), 4517. 10.3390/ijms23094517 35562908 PMC9101150

[B26] Moher D. Liberati A. Tetzlaff J. Altman D. G. Group P. (2009). Preferred reporting items for systematic reviews and meta-analyses: the PRISMA statement. J. Clin. Epidemiol. 62 (10), 1006–1012. 10.1016/j.jclinepi.2009.06.005 19631508

[B27] Nishio M. Barlesi F. West H. Ball S. Bordoni R. Cobo M. (2021). Atezolizumab plus chemotherapy for first-line treatment of nonsquamous NSCLC: Results from the randomized phase 3 IMpower132 trial. J. Thorac. Oncol. 16 (4), 653–664. 10.1016/j.jtho.2020.11.025 33333328

[B28] Owonikoko T. K. Park K. Govindan R. Ready N. Reck M. Peters S. (2021). Nivolumab and ipilimumab as maintenance therapy in extensive-disease small-cell lung cancer: CheckMate 451. J. Clin. Oncol. 39 (12), 1349–1359. 10.1200/JCO.20.02212 33683919 PMC8078251

[B29] Pardoll D. M. (2012). The blockade of immune checkpoints in cancer immunotherapy. Nat. Rev. Cancer 12 (4), 252–264. 10.1038/nrc3239 22437870 PMC4856023

[B30] Park S. K. Cho L. Y. Yang J. J. Park B. Chang S. H. Lee K. S. (2010). Lung cancer risk and cigarette smoking, lung tuberculosis according to histologic type and gender in a population based case-control study. Lung Cancer 68 (1), 20–26. 10.1016/j.lungcan.2009.05.017 19545930

[B31] Park K. Ozguroglu M. Vansteenkiste J. Spigel D. Yang J. C. H. Ishii H. (2021). Avelumab versus docetaxel in patients with platinum-treated advanced NSCLC: 2-Year follow-up from the JAVELIN lung 200 phase 3 trial. J. Thorac. Oncol. 16 (8), 1369–1378. 10.1016/j.jtho.2021.03.009 33845211

[B32] Paz-Ares L. Luft A. Vicente D. Tafreshi A. Gumus M. Mazieres J. (2018). Pembrolizumab plus chemotherapy for squamous non-small-cell lung cancer. N. Engl. J. Med. 379 (21), 2040–2051. 10.1056/NEJMoa1810865 30280635

[B33] Peters S. Pujol J. L. Dafni U. Domine M. Popat S. Reck M. (2022). Consolidation nivolumab and ipilimumab versus observation in limited-disease small-cell lung cancer after chemo-radiotherapy - results from the randomised phase II ETOP/IFCT 4-12 STIMULI trial. Ann. Oncol. 33 (1), 67–79. 10.1016/j.annonc.2021.09.011 34562610

[B34] Reck M. Luft A. Szczesna A. Havel L. Kim S. W. Akerley W. (2016). Phase III randomized trial of ipilimumab plus etoposide and platinum versus placebo plus etoposide and platinum in extensive-stage small-cell lung cancer. J. Clin. Oncol. 34 (31), 3740–3748. 10.1200/JCO.2016.67.6601 27458307

[B35] Reck M. Rodriguez-Abreu D. Robinson A. G. Hui R. Csoszi T. Fulop A. (2019). Updated analysis of KEYNOTE-024: Pembrolizumab versus platinum-based chemotherapy for advanced non-small-cell lung cancer with PD-L1 tumor proportion score of 50% or greater. J. Clin. Oncol. 37 (7), 537–546. 10.1200/JCO.18.00149 30620668

[B36] Reck M. Ciuleanu T. E. Cobo M. Schenker M. Zurawski B. Menezes J. (2021). First-line nivolumab plus ipilimumab with two cycles of chemotherapy versus chemotherapy alone (four cycles) in advanced non-small-cell lung cancer: CheckMate 9LA 2-year update. ESMO Open 6 (5), 100273. 10.1016/j.esmoop.2021.100273 34607285 PMC8493593

[B37] Rittmeyer A. Barlesi F. Waterkamp D. Park K. Ciardiello F. von Pawel J. (2017). Atezolizumab versus docetaxel in patients with previously treated non-small-cell lung cancer (OAK): a phase 3, open-label, multicentre randomised controlled trial. Lancet 389 (10066), 255–265. 10.1016/s0140-6736(16)32517-x 27979383 PMC6886121

[B38] Rodriguez-Abreu D. Powell S. F. Hochmair M. J. Gadgeel S. Esteban E. Felip E. (2021). Pemetrexed plus platinum with or without pembrolizumab in patients with previously untreated metastatic nonsquamous NSCLC: Protocol-specified final analysis from KEYNOTE-189. Ann. Oncol. 32 (7), 881–895. 10.1016/j.annonc.2021.04.008 33894335

[B39] Rudin C. M. Awad M. M. Navarro A. Gottfried M. Peters S. Csoszi T. (2020). Pembrolizumab or placebo plus etoposide and platinum as first-line therapy for extensive-stage small-cell lung cancer: Randomized, double-blind, phase III KEYNOTE-604 study. J. Clin. Oncol. 38 (21), 2369–2379. 10.1200/JCO.20.00793 32468956 PMC7474472

[B40] Sethi J. Ali M. S. Mohananey D. Nanchal R. Maldonado F. Musani A. (2019). Are transbronchial cryobiopsies ready for prime time?: A systematic review and meta-analysis. J. Bronchology Interv. Pulmonol. 26 (1), 22–32. 10.1097/LBR.0000000000000519 29901533

[B41] Sezer A. Kilickap S. Gümüş M. Bondarenko I. Özgüroğlu M. Gogishvili M. (2021). Cemiplimab monotherapy for first-line treatment of advanced non-small-cell lung cancer with PD-L1 of at least 50%: a multicentre, open-label, global, phase 3, randomised, controlled trial. Lancet 397 (10274), 592–604. 10.1016/s0140-6736(21)00228-2 33581821

[B42] Sibiya M. A. Raphoko L. Mangokoana D. Makola R. Nxumalo W. Matsebatlela T. M. (2019). Induction of cell death in human A549 cells using 3-(Quinoxaline-3-yl) prop-2-ynyl methanosulphonate and 3-(Quinoxaline-3-yl) prop-2-yn-1-ol. Molecules 24 (3), E407. 10.3390/molecules24030407 30678061 PMC6384999

[B43] Siegel R. L. Miller K. D. Fuchs H. E. Jemal A. (2022). Cancer statistics, 2022. CA. Cancer J. Clin. 72 (1), 7–33. 10.3322/caac.21708 35020204

[B44] Socinski M. A. Nishio M. Jotte R. M. Cappuzzo F. Orlandi F. Stroyakovskiy D. (2021). IMpower150 final overall survival analyses for atezolizumab plus bevacizumab and chemotherapy in first-line metastatic nonsquamous NSCLC. J. Thorac. Oncol. 16 (11), 1909–1924. 10.1016/j.jtho.2021.07.009 34311108

[B45] Song P. Zhang J. Shang C. Zhang L. (2019). Real-world evidenceand clinical observations of the treatment of advanced non-small cell lung cancer with PD-1/PD-L1 inhibitors. Sci. Rep. 9 (1), 4278. 10.1038/s41598-019-40748-7 30862891 PMC6414649

[B46] Spigel D. R. Vicente D. Ciuleanu T. E. Gettinger S. Peters S. Horn L. (2021). Second-line nivolumab in relapsed small-cell lung cancer: CheckMate 331. Ann. Oncol. 32 (5), 631–641. 10.1016/j.annonc.2021.01.071 33539946

[B47] Spigel D. R. Faivre-Finn C. Gray J. E. Vicente D. Planchard D. Paz-Ares L. (2022). Five-year survival outcomes from the PACIFIC trial: Durvalumab after chemoradiotherapy in stage III non-small-cell lung cancer. J. Clin. Oncol. 40 (12), 1301–1311. 10.1200/JCO.21.01308 35108059 PMC9015199

[B48] Sugawara S. Lee J. S. Kang J. H. Kim H. R. Inui N. Hida T. (2021). Nivolumab with carboplatin, paclitaxel, and bevacizumab for first-line treatment of advanced nonsquamous non-small-cell lung cancer. Ann. Oncol. 32 (9), 1137–1147. 10.1016/j.annonc.2021.06.004 34139272

[B49] Tian Y. Zhai X. Yan W. Zhu H. Yu J. (2021). Clinical outcomes of immune checkpoint blockades and the underlying immune escape mechanisms in squamous and adenocarcinoma NSCLC. Cancer Med. 10 (1), 3–14. 10.1002/cam4.3590 33230935 PMC7826453

[B50] Tournoy K. G. Thomeer M. Germonpre P. Derijcke S. De Pauw R. Galdermans D. (2018). Does nivolumab for progressed metastatic lung cancer fulfill its promises? An efficacy and safety analysis in 20 general hospitals. Lung Cancer 115, 49–55. 10.1016/j.lungcan.2017.11.008 29290261

[B51] Tumeh P. C. Hellmann M. D. Hamid O. Tsai K. K. Loo K. L. Gubens M. A. (2017). Liver metastasis and treatment outcome with anti-PD-1 monoclonal antibody in patients with melanoma and NSCLC. Cancer Immunol. Res. 5 (5), 417–424. 10.1158/2326-6066.CIR-16-0325 28411193 PMC5749922

[B52] Wang J. C. Livingstone A. M. (2003). Cutting edge: CD4+ T cell help can be essential for primary CD8+ T cell responses *in vivo* . J. Immunol. 171 (12), 6339–6343. 10.4049/jimmunol.171.12.6339 14662830

[B53] Wang Y. Zhang Q. Chen C. Hu Y. Miao L. Zhou Y. (2021). Association of brain metastases with immune checkpoint inhibitors efficacy in advanced lung cancer: A systematic review and meta-analysis. Front. Oncol. 11, 721760. 10.3389/fonc.2021.721760 34956860 PMC8694212

[B54] West H. McCleod M. Hussein M. Morabito A. Rittmeyer A. Conter H. J. (2019). Atezolizumab in combination with carboplatin plus nab-paclitaxel chemotherapy compared with chemotherapy alone as first-line treatment for metastatic non-squamous non-small-cell lung cancer (IMpower130): a multicentre, randomised, open-label, phase 3 trial. Lancet. Oncol. 20 (7), 924–937. 10.1016/s1470-2045(19)30167-6 31122901

[B55] Wu Y. Lin L. Shen Y. Wu H. (2018a). Comparison between PD-1/PD-L1 inhibitors (nivolumab, pembrolizumab, and atezolizumab) in pretreated NSCLC patients: Evidence from a Bayesian network model. Int. J. Cancer 143 (11), 3038–3040. 10.1002/ijc.31733 29987914

[B56] Wu Y. Ju Q. Jia K. Yu J. Shi H. Wu H. (2018b). Correlation between sex and efficacy of immune checkpoint inhibitors (PD-1 and CTLA-4 inhibitors). Int. J. Cancer 143 (1), 45–51. 10.1002/ijc.31301 29424425

[B57] Wu Y. L. Zhang L. Fan Y. Zhou J. Zhang L. Zhou Q. (2021). Randomized clinical trial of pembrolizumab vs chemotherapy for previously untreated Chinese patients with PD-L1-positive locally advanced or metastatic non-small-cell lung cancer: KEYNOTE-042 China study. Int. J. Cancer 148 (9), 2313–2320. 10.1002/ijc.33399 33231285 PMC8048589

[B58] Wu Y. Yang S. Ma J. Chen Z. Song G. Rao D. (2022). Spatiotemporal immune landscape of colorectal cancer liver metastasis at single-cell level. Cancer Discov. 12 (1), 134–153. 10.1158/2159-8290.CD-21-0316 34417225

[B59] Xu Y. Wang Q. Xie J. Chen M. Liu H. Zhan P. (2021). The predictive value of clinical and molecular characteristics or immunotherapy in non-small cell lung cancer: A meta-analysis of randomized controlled trials. Front. Oncol. 11, 732214. 10.3389/fonc.2021.732214 34557415 PMC8453160

[B60] Xue C. Zheng S. Dong H. Lu X. Zhang X. Zhang J. (2021). Association between efficacy of immune checkpoint inhibitors and sex: An updated meta-analysis on 21 trials and 12, 675 non-small cell lung cancer patients. Front. Oncol. 11, 627016. 10.3389/fonc.2021.627016 34513654 PMC8427763

[B61] Yan X. Tian X. Wu Z. Han W. (2020). Impact of age on the efficacy of immune checkpoint inhibitor-based combination therapy for non-small-cell lung cancer: A systematic review and meta-analysis. Front. Oncol. 10, 1671. 10.3389/fonc.2020.01671 33072551 PMC7538697

[B62] Yang H. Ma W. Sun B. Fan L. Xu K. Hall S. R. R. (2021). Smoking signature is superior to programmed death-ligand 1 expression in predicting pathological response to neoadjuvant immunotherapy in lung cancer patients. Transl. Lung Cancer Res. 10 (9), 3807–3822. 10.21037/tlcr-21-734 34733630 PMC8512473

[B63] Yong T. Chang K. K. Wang Y. S. Ma C. (2022). Active humoral response reverts tumorigenicity through disruption of key signaling pathway. Vaccines (Basel) 10 (2), 163. 10.3390/vaccines10020163 35214622 PMC8875535

[B64] Yu X. Zhang X. Yao T. Zhang Y. Zhang Y. (2021). Fatal adverse events associated with immune checkpoint inhibitors in non-small cell lung cancer: a systematic review and meta-analysis. Front. Med. (Lausanne) 8, 627089. 10.3389/fmed.2021.627089 33659263 PMC7917063

