# Supplementary material for: The influence of baseline characteristics on the efficacy of immune checkpoint inhibitors for advanced lung cancer: A systematic review and meta-analysis
Source: Front Pharmacol. 2022 Sep 9;13:956788. doi: 10.3389/fphar.2022.956788 (PMC9513719; doi:10.3389/fphar.2022.956788)
Supplement: Supplementary file 1 [file DataSheet1.docx]

Supplementary Material

# Supplementary Figures and Tables

## Supplementary Figures

##
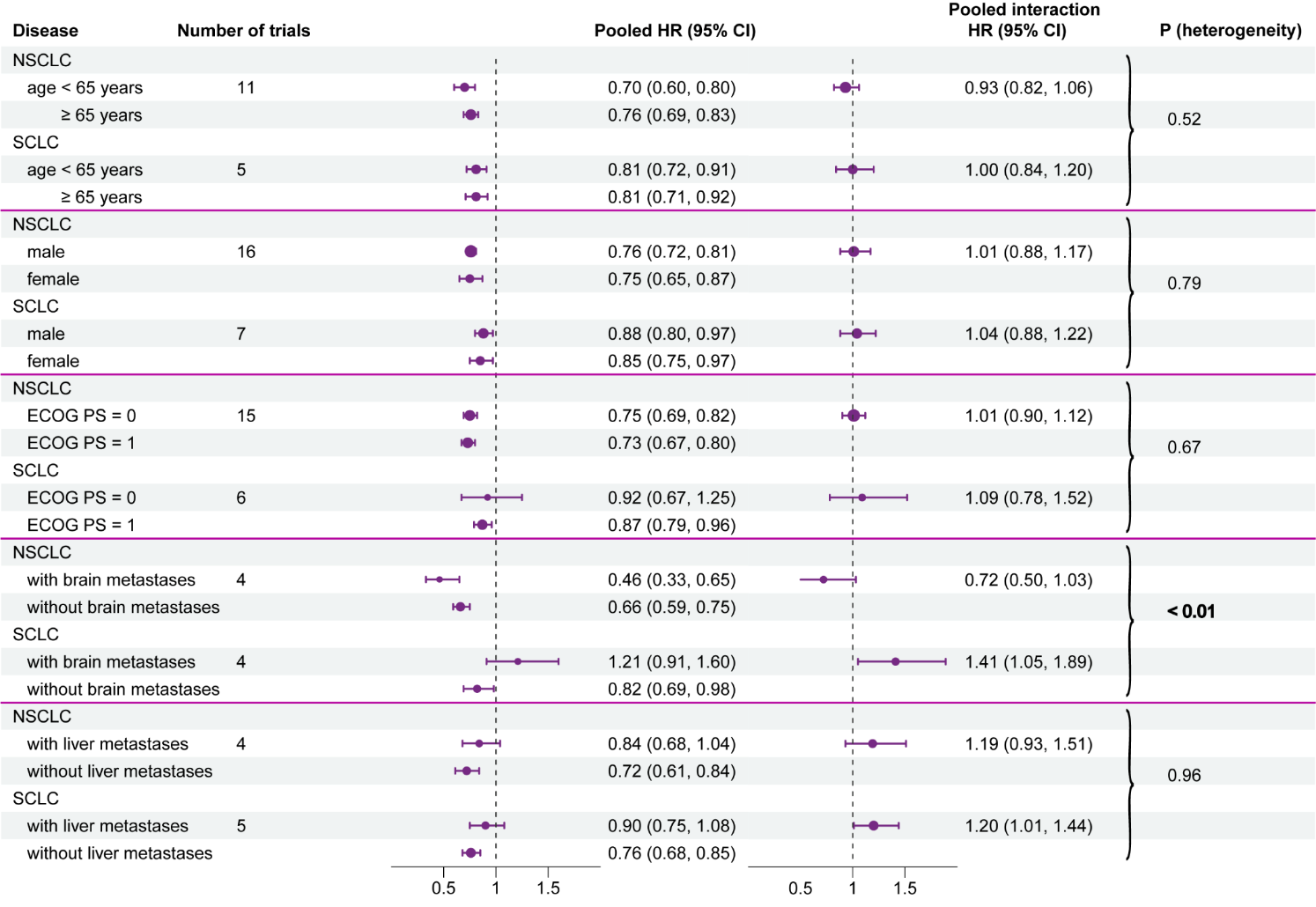


**Supplementary Figure S1.** Forest plot of hazard ratios for overall survival according to the subgroup of pathological type. The left forest plot: hazard ratios of overall survival for the subgroup and baseline characteristics. The right forest plot: interaction between immunotherapy efficacy and baseline characteristics in the subgroup.


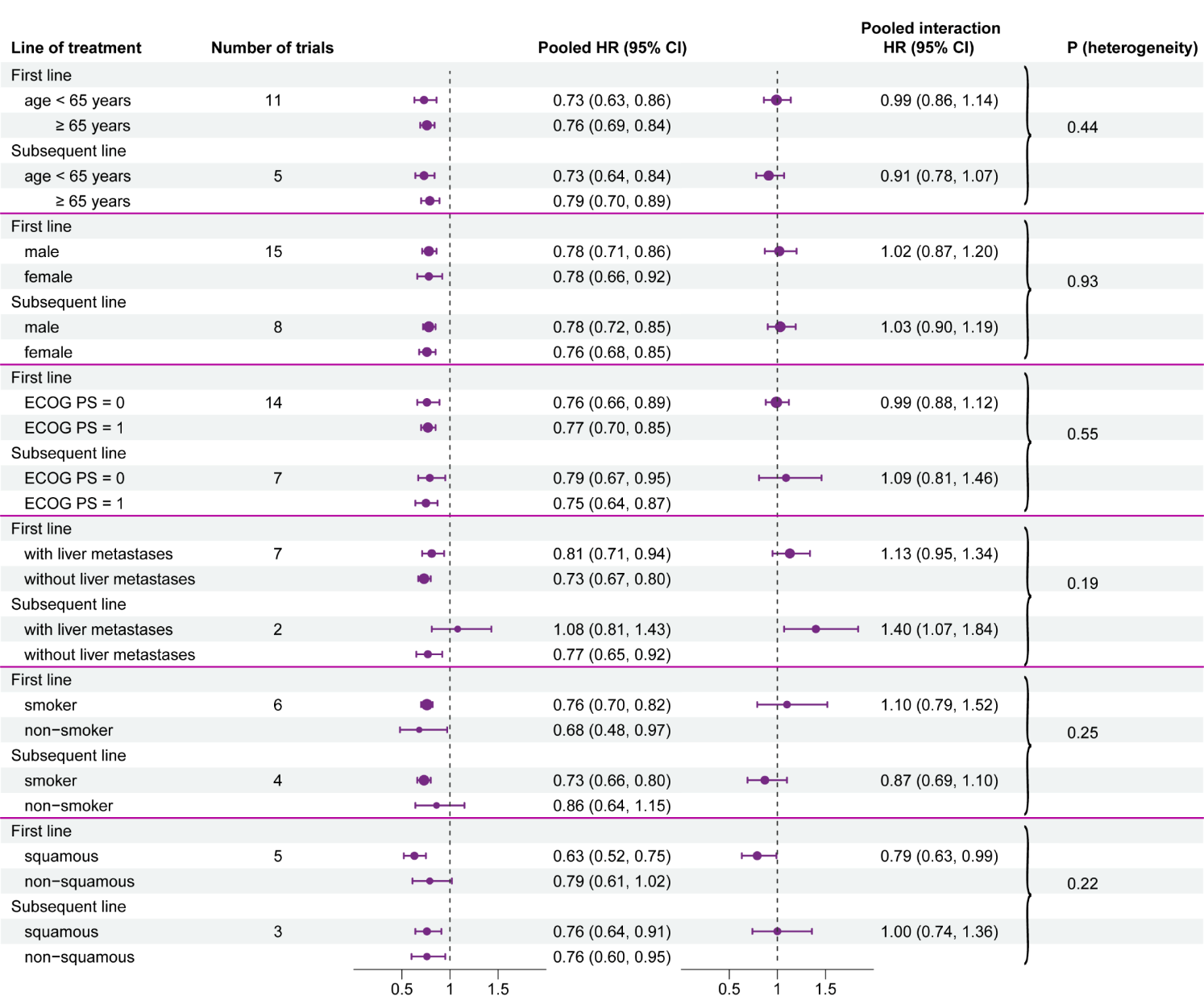


**Supplementary Figure S2.** Forest plot of hazard ratios for overall survival according to the subgroup of line of treatment. The left forest plot: hazard ratios of overall survival for the subgroup and baseline characteristics. The right forest plot: interaction between immunotherapy efficacy and baseline characteristics in subgroups.


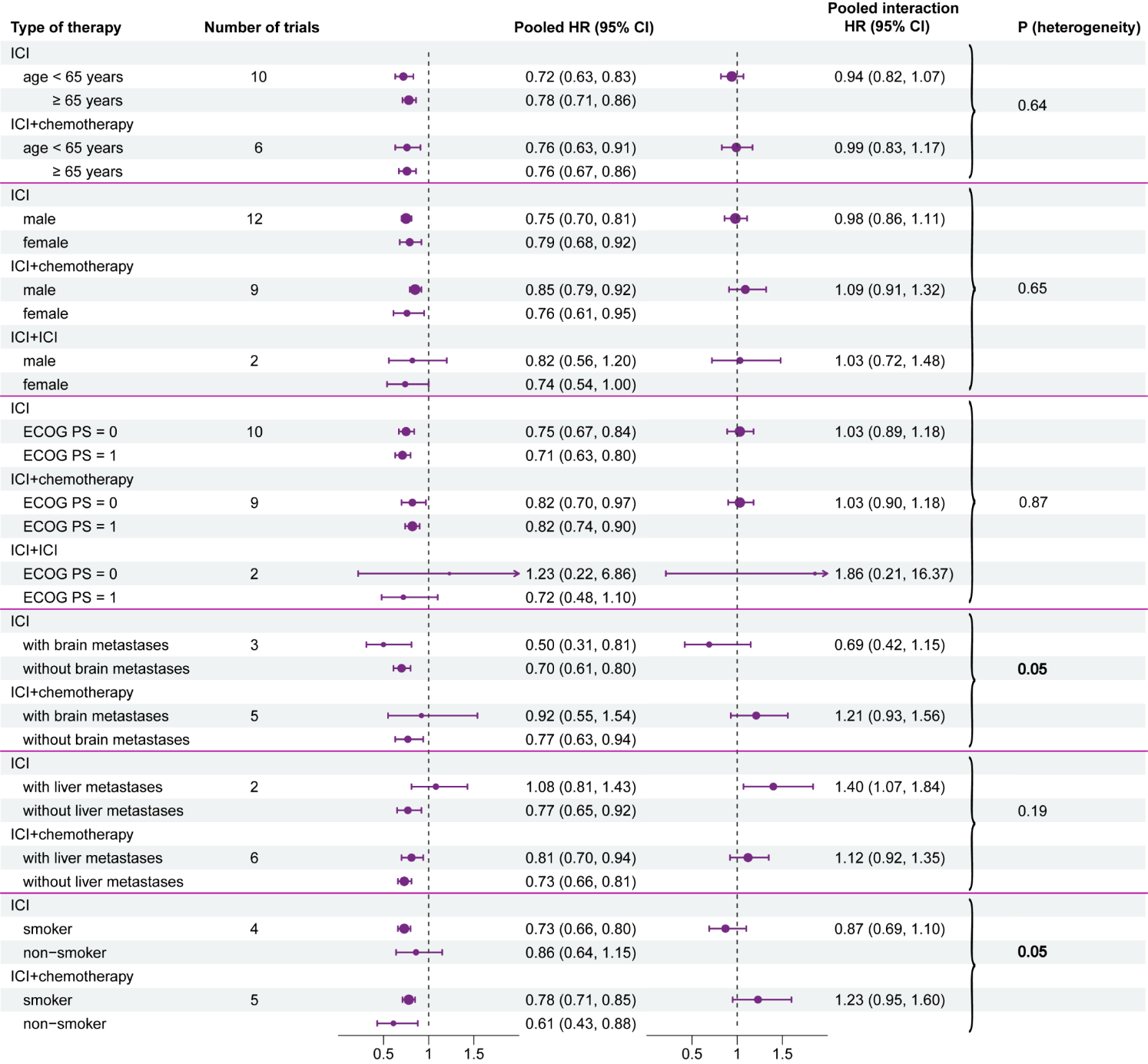


**Supplementary Figure S3.** Forest plot of hazard ratios for overall survival according to the subgroup of type of therapy. The left forest plot: hazard ratios of overall survival for the subgroup and baseline characteristics. The right forest plot: interaction between immunotherapy efficacy and baseline characteristics in the subgroup.


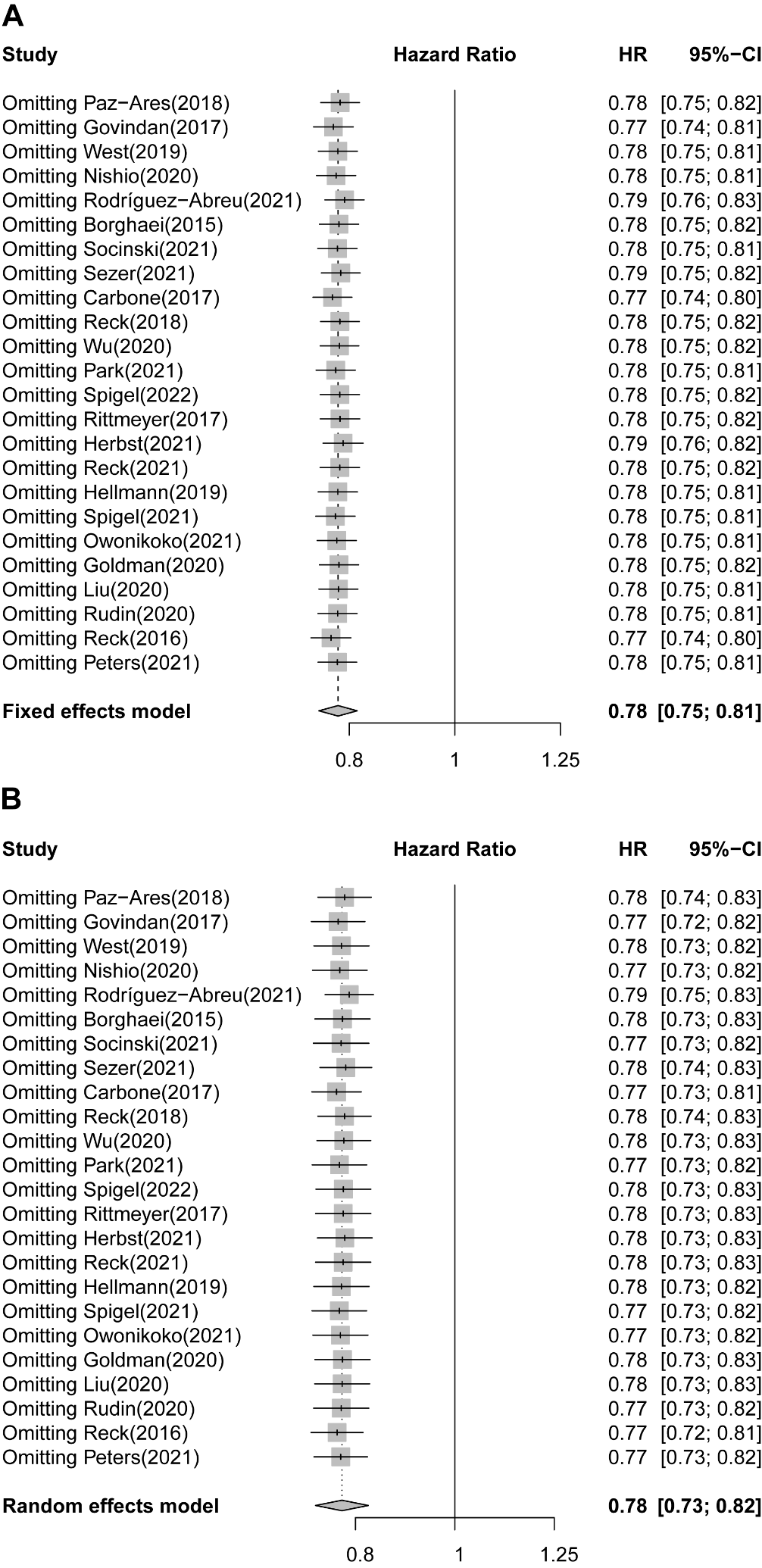


**Supplementary Figure S4.** Sensitivity analysis of hazard ratios for overall survival for all patients in included RCTs **(A)** Sensitivity analysis using the fixed effects model. **(B)** Sensitivity analysis using the random effects model.


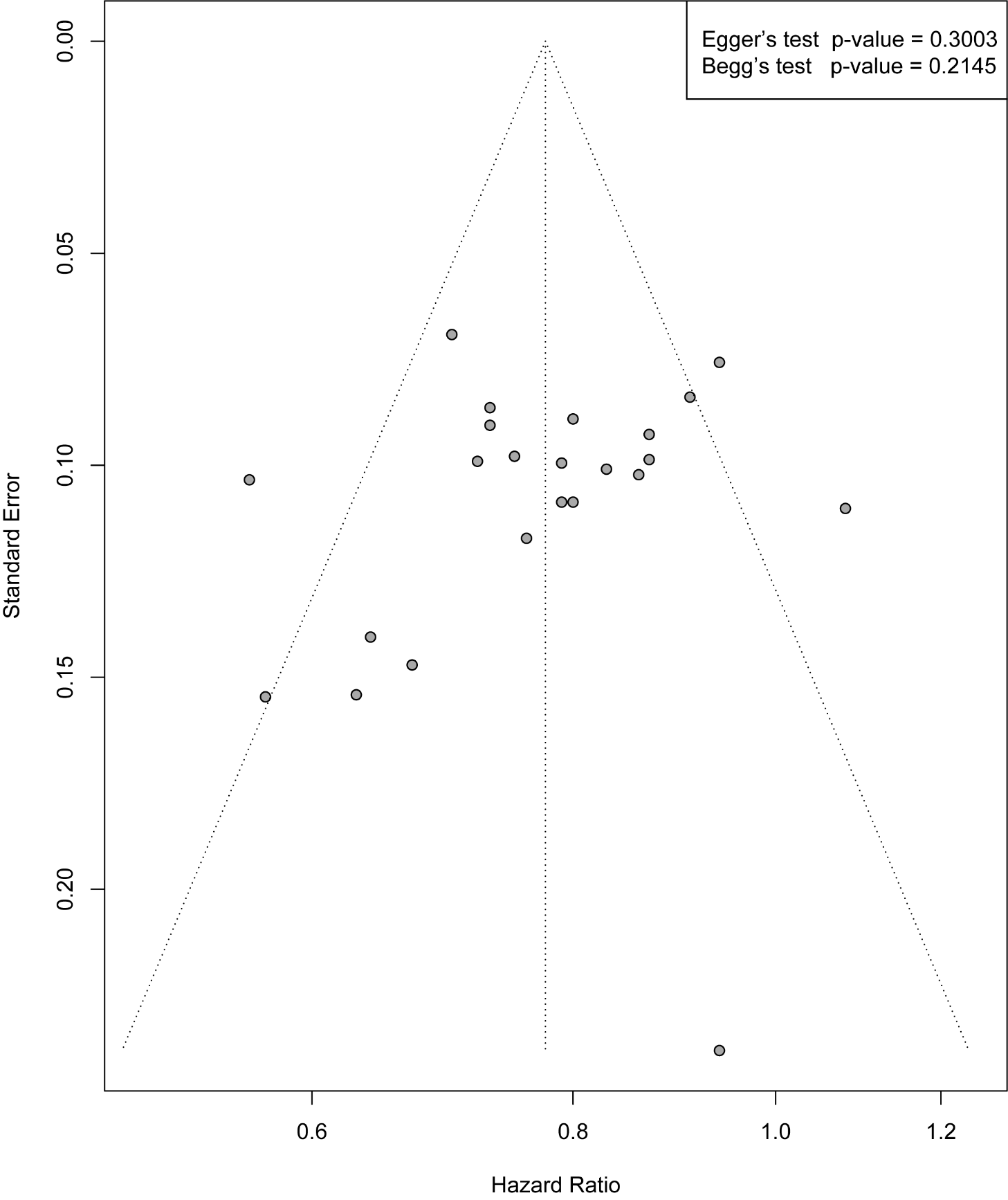


**Supplementary Figure S5.** Funnel plot of studies evaluating the difference in efficacy between immunotherapy and control group.

## Supplementary Tables

**Supplementary Table S1.** PRISMA 2009 Checklist

| **Section/topic** | **#** | **Checklist item** | **Reported on page #** |
| --- | --- | --- | --- |
| **TITLE** | | |  |
| Title | 1 | Identify the report as a systematic review, meta-analysis, or both. | 1 |
| **ABSTRACT** | | |  |
| Structured summary | 2 | Provide a structured summary including, as applicable: background; objectives; data sources; study eligibility criteria, participants, and interventions; study appraisal and synthesis methods; results; limitations; conclusions and implications of key findings; systematic review registration number. | 1 |
| **INTRODUCTION** | | |  |
| Rationale | 3 | Describe the rationale for the review in the context of what is already known. | 2 |
| Objectives | 4 | Provide an explicit statement of questions being addressed with reference to participants, interventions, comparisons, outcomes, and study design (PICOS). | 2 |
| **METHODS** | | |  |
| Protocol and registration | 5 | Indicate if a review protocol exists, if and where it can be accessed (e.g., Web address), and, if available, provide registration information including registration number. | 2 |
| Eligibility criteria | 6 | Specify study characteristics (e.g., PICOS, length of follow-up) and report characteristics (e.g., years considered, language, publication status) used as criteria for eligibility, giving rationale. | 2-3 |
| Information sources | 7 | Describe all information sources (e.g., databases with dates of coverage, contact with study authors to identify additional studies) in the search and date last searched. | 2 |
| Search | 8 | Present full electronic search strategy for at least one database, including any limits used, such that it could be repeated. | 2 |
| Study selection | 9 | State the process for selecting studies (i.e., screening, eligibility, included in systematic review, and, if applicable, included in the meta-analysis). | 3 |
| Data collection process | 10 | Describe method of data extraction from reports (e.g., piloted forms, independently, in duplicate) and any processes for obtaining and confirming data from investigators. | 3 |
| Data items | 11 | List and define all variables for which data were sought (e.g., PICOS, funding sources) and any assumptions and simplifications made. | 3 |
| **Section/topic** | **#** | **Checklist item** | **Reported on page #** |
| Risk of bias in individual studies | 12 | Describe methods used for assessing risk of bias of individual studies (including specification of whether this was done at the study or outcome level), and how this information is to be used in any data synthesis. | 3 |
| Summary measures | 13 | State the principal summary measures (e.g., risk ratio, difference in means). | 3 |
| Synthesis of results | 14 | Describe the methods of handling data and combining results of studies, if done, including measures of consistency (e.g., I^2^) for each meta-analysis. | 3 |
| Risk of bias across studies | 15 | Specify any assessment of risk of bias that may affect the cumulative evidence (e.g., publication bias, selective reporting within studies). | 3 |
| Additional analyses | 16 | Describe methods of additional analyses (e.g., sensitivity or subgroup analyses, meta-regression), if done, indicating which were pre-specified. | 3 |
| **RESULTS** | | |  |
| Study selection | 17 | Give numbers of studies screened, assessed for eligibility, and included in the review, with reasons for exclusions at each stage, ideally with a flow diagram. | 3 |
| Study characteristics | 18 | For each study, present characteristics for which data were extracted (e.g., study size, PICOS, follow-up period) and provide the citations. | 3 |
| Risk of bias within studies | 19 | Present data on risk of bias of each study and, if available, any outcome level assessment (see item 12). | 3 |
| Results of individual studies | 20 | For all outcomes considered (benefits or harms), present, for each study: (a) simple summary data for each intervention group (b) effect estimates and confidence intervals, ideally with a forest plot. | 4 |
| Synthesis of results | 21 | Present results of each meta-analysis done, including confidence intervals and measures of consistency. | 4 |
| Risk of bias across studies | 22 | Present results of any assessment of risk of bias across studies (see Item 15). | 3 |
| Additional analysis | 23 | Give results of additional analyses, if done (e.g., sensitivity or subgroup analyses, meta-regression [see Item 16]). | 6 |
| **DISCUSSION** | | |  |
| Summary of evidence | 24 | Summarize the main findings including the strength of evidence for each main outcome; consider their relevance to key groups (e.g., healthcare providers, users, and policy makers). | 7-10 |
| Limitations | 25 | Discuss limitations at study and outcome level (e.g., risk of bias), and at review-level (e.g., incomplete retrieval of identified research, reporting bias). | 10 |
| Conclusions | 26 | Provide a general interpretation of the results in the context of other evidence, and implications for future research. | 10 |
| **FUNDING** | | |  |
| Funding | 27 | Describe sources of funding for the systematic review and other support (e.g., supply of data); role of funders for the systematic review. | 10 |

**Supplementary Table S2.** The details of the search strategy.

| **Pubmed** |
| --- |
| ((((((((((((((((((((((((Nivolumab[MeSH Terms]) OR (nivolumab)) OR (Opdivo)) OR (ONO-4538)) OR (ONO 4538)) OR (ONO4538)) OR (MDX-1106)) OR (MDX 1106)) OR (MDX1106)) OR (BMS-936558)) OR (BMS 936558)) OR (BMS936558)) OR  ((((((Pembrolizumab[MeSH Terms]) OR (pembrolizumab)) OR (SCH-900475)) OR (Keytruda)) OR (MK-3475)) OR (lambrolizumab))) OR  (((Cemiplimab[MeSH Terms]) OR (cemiplimab)) OR (REGN2810))) OR  ((((((Programmed Cell Death 1 Receptor[MeSH Terms]) OR (PD-1)) OR (PD 1)) OR (PD 1 Receptor)) OR (PD-1 Receptor)) OR (CD279 Antigen))) OR  (((((((Atezolizumab[MeSH Terms]) OR (atezolizumab)) OR (MPDL3280A)) OR (MPDL-3280A)) OR (Tecentriq)) OR (RG7446)) OR (RG-7446))) OR  (((((Durvalumab[MeSH Terms]) OR (durvalumab)) OR (MEDI4736)) OR (MEDI-4736)) OR (Imfinzi))) OR  (((((((Avelumab[MeSH Terms]) OR (avelumab)) OR (MSB-0010682)) OR (MSB0010682)) OR (bavencio)) OR (MSB0010718C)) OR (MSB-0010718C))) OR  ((((((B7-H1 Antigen[MeSH Terms]) OR (PD-L1)) OR (PDL1)) OR (PD L1)) OR (Programmed Cell Death 1 Ligand 1 Protein)) OR (CD274 Antigens))) OR  ((((((((Ipilimumab[MeSH Terms]) OR (ipilimumab)) OR (Yervoy)) OR (MDX 010)) OR (MDX010)) OR (MDX-010)) OR (MDX-CTLA-4)) OR (MDX CTLA 4))) OR  ((((((((((Tremelimumab[MeSH Terms]) OR (tremelimumab)) OR (ticilimumab)) OR (CP 675)) OR (CP675 cpd)) OR (CP-675)) OR (CP-675,206)) OR (CP-675206)) OR (CP675206)) OR (CP 675206))) OR  ((((((CTLA-4 Antigen[MeSH Terms]) OR (CTLA-4)) OR (CTLA 4)) OR (Cytotoxic T-Lymphocyte Associated Antigen 4)) OR (Cytotoxic T-Lymphocyte Antigen 4)) OR (CD152 Antigen))) OR (immune checkpoint inhibitor)) AND  ((((((((((((((((((((Lung Neoplasms[MeSH Terms]) OR (Pulmonary Neoplasms)) OR (Neoplasms, Lung)) OR (Lung Neoplasm)) OR (Neoplasm, Lung)) OR (Neoplasms, Pulmonary)) OR (Neoplasm, Pulmonary)) OR (Pulmonary Neoplasm)) OR (Lung Cancer)) OR (Cancer, Lung)) OR (Cancers, Lung)) OR (Lung Cancers)) OR (Pulmonary Cancer)) OR (Cancer, Pulmonary)) OR (Cancers, Pulmonary)) OR (Pulmonary Cancers)) OR (Cancer of the Lung)) OR (Cancer of Lung)) OR (NSCLC)) OR (SCLC))) AND  ((randomized controlled trial[Publication Type]) OR (clinical trial[Publication Type])) |
| **Embase** |
| #1 'lung tumor'/exp  #2 'lung cancer'  #3 'lung carcinoma'  #4 NSCLC  #5 SCLC  #6 #1 OR #2 OR #3 OR #4 OR #5  #7 'pembrolizumab'/exp  #8 'pembrolizumab'  #9 keytruda  #10 'lambrolizumab'  #11 'mk 3475' OR 'mk3475' OR 'sch 900475' OR 'sch900475'  #12 'nivolumab'/exp  #13 'nivolumab'  #14 opdivo  #15 'bms 936558' OR 'bms936558' OR 'cmab 819' OR 'cmab819' OR 'mdx 1106' OR 'mdx1106' OR 'ono 4538' OR 'ono4538'  #16 'Cemiplimab'/exp  #17'Cemiplimab'  #18 'cemiplimab rwlc' OR 'cemiplimab-rwlc' OR 'libtayo' OR 'regn 2810' OR 'regn2810' OR 'sar 439684' OR 'sar439684'  #19 'programmed death 1 receptor'/exp  #20 'Programmed Cell Death 1 Receptor' OR 'PD-1' OR 'PD 1' OR 'PD 1 Receptor' OR 'PD-1 Receptor' OR 'CD279 Antigen'  #21 'atezolizumab'/exp  #22 'atezolizumab'  #23 tecentriq  #24 'monoclonal antibody mpdl 3280a' OR 'monoclonal antibody mpdl3280a' OR 'mpdl 3280a' OR 'mpdl3280a' OR 'rg 7446' OR 'rg7446' OR 'ro 5541267' OR 'ro5541267' OR 'tecntriq'  #25 'durvalumab'/exp  #26 'durvalumab'  #27 imfinzi  #28 'medi 4736' OR 'medi4736'  #29 'avelumab'/exp  #30 'avelumab'  #31 bavencio  #32 'msb 0010682' OR 'msb 0010718c' OR 'msb 10682' OR 'msb 10718c' OR 'msb0010682' OR 'msb0010718c' OR 'msb10682' OR 'msb10718c' OR 'pf 06834635' OR 'pf 6834635' OR 'pf06834635' OR 'pf6834635'  #33 'programmed death 1 ligand 1'/exp  #34 'PD-L1' OR 'PDL1' OR 'PD L1' OR 'Programmed Cell Death 1 Ligand 1 Protein' OR 'CD274 Antigens'  #35 'ipilimumab'/exp  #36 'ipilimumab'  #37 yervoy  #38 'bms 734016' OR 'bms734016' OR 'cs 1002' OR 'cs1002' OR 'ibi 310' OR 'ibi310' OR 'mdx 010' OR 'mdx 101' OR 'mdx010' OR 'mdx101' OR 'strentarga'  #39 'tremelimumab'/exp  #40 'tremelimumab'  #41 'ticilimumab'  #42 'cp 675 206' OR 'cp 675, 206' OR 'cp 675206' OR 'cp675 206' OR 'cp675, 206' OR 'cp675206'  #43 'cytotoxic T lymphocyte antigen 4'/exp  #44 'CTLA-4 Antigen' OR 'CTLA-4' OR 'CTLA 4' OR 'Cytotoxic T-Lymphocyte Associated Antigen 4' OR 'Cytotoxic T-Lymphocyte Antigen 4' OR 'CD152 Antigen'  #45 'immune checkpoint inhibitor*'  #46 #7 OR #8 OR #9 OR #10 OR #11 OR #12 OR #13 OR #14 OR #15 OR #16 OR #17 OR #18 OR #19 OR #20 OR #21 OR #22 OR #23 OR #24 OR #25 OR #26 OR #27 OR #28 OR #29 OR #30 OR #31 OR #32 OR #33 OR #34 OR #35 OR #36 OR #37 OR #38 OR #39 OR #40 OR #41 OR #42 OR #43 OR #44 OR #45  #47 #6 AND #46 |
| **Cochrane Library** |
| #1 MeSH descriptor: [Lung Neoplasms] explode all trees  #2 Lung Cancer  #3 NSCLC  #4 SCLC  #5 #1 OR #2 OR #3 OR #4  #6 MeSH descriptor: [Nivolumab] explode all trees  #7 Nivolumab  #8 Opdivo  #9 BMS 936558  #10 BMS-936558  #11 BMS936558  #12 MDX 1106  #13 MDX-1106  #14 ONO 4538  #15 ONO-4538  #16 #6 OR #7 OR #8 OR #9 OR #10 OR #11 OR #12 OR #13 OR #14 OR #15  #17 Pembrolizumab  #18 Keytruda  #19 Lambrolizumab  #20 Merck 3475  #21 MK 3475  #22 MK-3475  #23 MK3475  #24 Sch 900475  #25 SCH-900475  #26 #17 OR #18 OR #19 OR #20 OR #21 OR #22 OR #23 OR #24 OR #25  #27 Cemiplimab  #28 REGN2810  #29 #27 OR #28  #30 MeSH descriptor: [Programmed Cell Death 1 Receptor] explode all trees  #31 Programmed Cell Death 1 Protein  #32 Programmed Cell Death Protein 1  #33 PD1 Receptor  #34 PD 1 Receptor  #35 PD-1 Receptor  #36 CD279 Antigen  #37 #30 OR #31 OR #32 OR #33 OR #34 OR #35 OR #36  #38 Atezolizumab  #39 Tecentriq  #40 MPDL 3280A  #41 MPDL-3280A  #42 MPDL3280A  #43 RG-7446  #44 RG7446  #45 #38 OR #39 OR #40 OR #41 OR #42 OR #43 OR #44  #46 Durvalumab  #47 Imfinzi  #48 MEDI 4736  #49 MEDI-4736  #50 MEDI4736  #51 #46 OR #47 OR #48 OR #49 OR #50  #52 Avelumab  #53 Bavencio  #54 MSB-0010682  #55 MSB0010682  #56 MSB0010718C  #57 MSB-0010718C  #58 #52 OR #53 OR #54 OR #55 OR #56 OR #57  #59 MeSH descriptor: [B7-H1 Antigen] explode all trees  #60 PD-L1  #61 PDL1  #62 PD L1  #63 Programmed Cell Death 1 Ligand 1 Protein  #64 CD274 Antigens  #65 Costimulatory Protein  #66 Immune Costimulatory Protein  #67 #59 OR #60 OR #61 OR #62 OR #63 OR #64 OR #65 OR #66  #68 MeSH descriptor: [Ipilimumab] explode all trees  #69 Ipilimumab  #70 Yervoy  #71 BMS-734016  #72 MDX-010  #73 MDX-101  #74 MDX-CTLA-4  #75 MOAB-CTLA-4  #76 #68 OR #69 OR #70 OR #71 OR #72 OR #73 OR #74 OR #75  #77 Tremelimumab  #78 Ticilimumab  #79 CP675 cpd  #80 CP-675  #81 CP-675,206  #82 CP-675206  #83 #77 OR #78 OR #79 OR #80 OR #81 OR #82  #84 MeSH descriptor: [CTLA-4 Antigen] explode all trees  #85 CTLA-4 Antigen  #86 CTLA-4  #87 CTLA 4  #88 Cytotoxic T-Lymphocyte Associated Antigen 4  #89 Cytotoxic T-Lymphocyte Antigen 4  #90 CD152 Antigen  #91 #84 OR #85 OR #86 OR #87 OR #88 OR #89 OR #90  #92 immune checkpoint inhibitor*  #93 #16 OR #26 OR #29 OR #37 OR #45 OR #51 OR #58 OR #67 OR #76 OR #83 OR #91 OR # 92  #94 #5 AND #93 |

**Supplementary Table S3.** The methodological quality assessment to 24 included trials.

| First author | Study ID | Random Sequence Generation | Allocation Concealment | Blinding of Participants and Personnel | Blinding of Outcome Assessment | Incomplete Outcome Data | Selective Reporting | Other Bias |
| --- | --- | --- | --- | --- | --- | --- | --- | --- |
| Paz-Ares et al. (2018) | KEYNOTE-407 | Low Risk | Low Risk | Low Risk | Unclear Risk | Low Risk | Low Risk | Low Risk |
| Borghaei et al. (2015) | CheckMate 057 | Low Risk | High Risk | High Risk | High Risk | Low Risk | Low Risk | Low Risk |
| Sezer et al.(2021) | EMPOWER-Lung 1 | Low Risk | Unclear Risk | Unclear Risk | High Risk | Low Risk | Low Risk | Low Risk |
| Carbone et al. (2017) | CheckMate 026 | Low Risk | High Risk | High Risk | High Risk | Low Risk | Low Risk | Low Risk |
| Reck et al.(2019) | KEYNOTE-024 | Low Risk | Unclear Risk | Unclear Risk | High Risk | Low Risk | Low Risk | Low Risk |
| Wu et al.(2021) | KEYNOTE-042 China Study | Low Risk | Unclear Risk | Unclear Risk | High Risk | Low Risk | Low Risk | Low Risk |
| Park et al.(2021) | JAVELIN Lung 200 | Low Risk | High Risk | High Risk | High Risk | Low Risk | Low Risk | Low Risk |
| Spigel et al.(2022) | PACIFIC | Low Risk | Low Risk | Low Risk | Low Risk | Low Risk | Low Risk | Low Risk |
| Rittmeyer et al. (2017) | OAK study | Low Risk | Unclear Risk | Unclear Risk | High Risk | Low Risk | Low Risk | Low Risk |
| Herbst et al.(2021) | KEYNOTE-010 | Low Risk | High Risk | High Risk | High Risk | Low Risk | Low Risk | Low Risk |
| Govindan et al. (2017) | NR | Low Risk | Low Risk | Low Risk | Unclear Risk | Low Risk | Low Risk | Low Risk |
| West et al.(2019) | IMpower130 | Low Risk | High Risk | High Risk | High Risk | Low Risk | Low Risk | Low Risk |
| Nishio et al.(2021) | IMpower132 | Low Risk | Unclear Risk | Unclear Risk | High Risk | Low Risk | Low Risk | Low Risk |
| Rodríguez-Abreu et al.(2021) | KEYNOTE-189 | Low Risk | Low Risk | Low Risk | Unclear Risk | Low Risk | Low Risk | Low Risk |
| Socinski et al. (2021) | IMpower150 | Low Risk | High Risk | High Risk | High Risk | Low Risk | Low Risk | Low Risk |
| Hellmann et al. (2019) | CheckMate 227 | Low Risk | Unclear Risk | Unclear Risk | High Risk | Low Risk | Low Risk | Low Risk |
| Reck et al.(2021) | CheckMate 9LA | Low Risk | High Risk | High Risk | High Risk | Low Risk | Low Risk | Low Risk |
| Spigel et al.(2021) | CheckMate 331 | Low Risk | Unclear Risk | Unclear Risk | High Risk | Low Risk | Low Risk | Low Risk |
| Owonikoko et al. (2021) | CheckMate 451 | Low Risk | Low Risk | Low Risk | Unclear Risk | Low Risk | Low Risk | Low Risk |
| Goldman et al. (2021) | CASPIAN | Low Risk | Unclear Risk | Unclear Risk | High Risk | Low Risk | Low Risk | Low Risk |
| Liu et al.(2021) | IMpower133 | Low Risk | Low Risk | Low Risk | Unclear Risk | Low Risk | Low Risk | Low Risk |
| Rudin et al.(2020) | KEYNOTE-604 | Low Risk | Low Risk | Low Risk | Unclear Risk | Low Risk | Low Risk | Low Risk |
| Reck et al.(2016) | NR | Low Risk | Low Risk | Low Risk | Unclear Risk | Low Risk | Low Risk | Low Risk |
| Peters et al.(2022) | STIMULI trial | Low Risk | High Risk | High Risk | High Risk | Low Risk | Low Risk | Low Risk |
